# Associations of Maternal Nutritional Status and Supplementation with Fetal, Newborn, and Infant Outcomes in Low-Income and Middle-Income Settings: An Overview of Reviews

**DOI:** 10.3390/nu16213725

**Published:** 2024-10-31

**Authors:** Doris González-Fernández, Oviya Muralidharan, Paulo A. Neves, Zulfiqar A. Bhutta

**Affiliations:** Centre for Global Child Health, Hospital for Sick Children, Toronto, ON M5G 0A4, Canada

**Keywords:** maternal under-nutrition, prenatal supplementation, intrauterine growth restriction, low birthweight, preterm birth, small for gestational age

## Abstract

Background/Objectives: Despite advances in maternal nutritional knowledge, the effect of maternal diet, micronutrient status and undernutrition, and the effect of maternal supplementation on fetal, neonatal and infant outcomes still have gaps in the literature. This overview of reviews is intended to assess the available information on these issues and identify the main maternal nutritional factors associated with offspring outcomes in low- and middle-income countries as possible targets for public health interventions. Methods: The literature search was performed in Medline (PubMed) and Cochrane Library datasets in June 2024. Pre-specified outcomes in offspring were pooled using standard meta-analytical methods. Results: We found consistent evidence on the impact of maternal undernutrition indicated by low body mass index (BMI), mid-upper arm circumference (MUAC), and stature, but not of individual micronutrient status, on intrauterine-growth retardation, preterm birth, low birth weight, and small for gestational age, with research showing a possible effect of maternal undernutrition in later child nutritional status. Studies on micronutrient supplementation showed possible beneficial effects of iron, vitamin D, and multiple micronutrients on birthweight and/or decreasing small for gestational age, as well as a possible effect of calcium on preterm birth reduction. Interventions showing more consistent beneficial outcomes were balanced protein-energy and lipid base supplements, which demonstrated improved weight in newborns from supplemented mothers and a decreased risk of adverse neonatal outcomes. Conclusions: Further research is needed to identify the benefits and risks of maternal individual micronutrient supplementation on neonatal and further child outcomes.

## 1. Introduction

Some progress has been observed towards the United Nations Agenda for Sustainable Development by 2030, including the Sustainable Development Goal (SDG) 2 (end hunger, achieve food security and improved nutrition, and promote sustainable agriculture) and SDG 3 (ensure healthy lives and promote well-being for all at all ages) [1]. For example, the prevalence of low body mass index among women of reproductive age has been halved in middle-income countries, but short stature and anemia remain unchanged in less developed areas of the world [2]. Levels of undernourishment globally rose sharply in 2020 and have stagnated since then. For countries to achieve SDG nutrition targets by 2030, malnutrition must be addressed through the life course lens, with the timing of nutrition interventions in each period, from pre-conception, pregnancy, and lactation through infancy, childhood, adolescence, adulthood, and older ages [3].

In 2019, child and maternal malnutrition were estimated to be responsible for around three million deaths [4]. In addition, 45% of deaths reported in children under the age of five have been attributed to undernutrition [5]. The burden of undernutrition is also disproportionately distributed, with 70% of all children under five affected by wasting estimated to live in Asia and more than one quarter living in Africa [6].

The problem of child undernutrition starts in utero, expressed as intrauterine growth restriction (IUGR), which refers to the inability of the fetus to achieve its genetic growth potential, mainly due to placental hypo-perfusion and/or inflammation, and leading to higher perinatal mortality, higher risk of adverse outcomes, and lifelong health consequences [7]. Low birth weight (LBW defined as birth weight <2500 g regardless of gestational age [1]), small for gestational age (SGA) (birth weight <10th percentile for the appropriate gestational age) [8], and preterm birth (PTB) (babies born before 37 weeks of pregnancy) [9] are also known to have wide consequences at short and long term, with costs related with health care provision during hospitalization, discharge, and follow up during childhood [10]. LBW babies have an increased risk of perinatal morbidity and mortality, with later effects on linear growth, metabolic and mental development, as well as an increased risk of disease later in life [11], particularly cardiovascular and renal disease [12].

Maternal nutritional status is a strong predictor of fetal growth and birth outcomes, and has been associated with IUGR and LBW [13]. Maternal undernutrition, defined as having a body mass index of <18.5, has intergenerational consequences, as it has been found to be an important determinant of child undernutrition [14], and its effects continue throughout the lifecycle, impacting developmental and economic outcomes [15]. Decreased growth from conception to two years of age influences further growth and health outcomes [16], disproportionally affecting LMICs. In South Asia, for example, maternal malnutrition alone accounts for 25–50% of IUGR [17].

**Figure 1:**
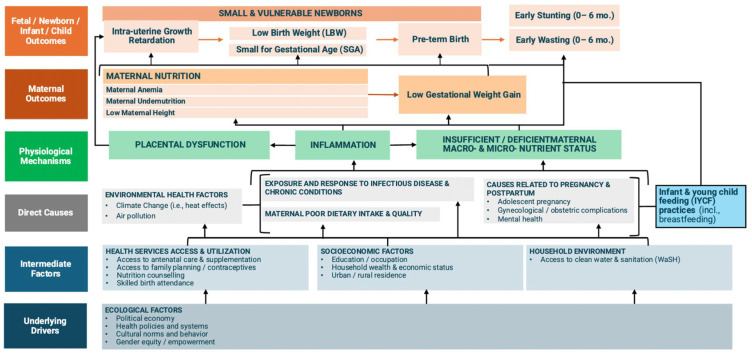
Environmental and maternal pathways known to affect fetal, newborn, and infant growth.

Evidence shows beneficial effects on child growth from interventions improving nutrition during pregnancy and early childhood, in contrast with smaller effects of interventions during childhood and those aiming to reduce infections, such as water, sanitation, and hygiene interventions [16]. Based on current knowledge, we developed a conceptual framework showing the complexity and multifactorial impact of environmental and maternal factors affecting fetal, neonatal, and infant outcomes (Figure 1). However, literature reviews usually combine evidence from high (HICs) and low- and middle-income countries (LMICs), making it difficult to clarify the applicability of research in resource-limited settings. It is also not clear how studies differentiate the impact of maternal nutritional status and nutritional interventions in the different stages of pregnancy on the offspring.

The objective of this overview of reviews is to understand better how maternal nutritional status during preconception, early pregnancy, late pregnancy, and the lactation period associates with offspring outcomes during fetal (growth restriction and death), neonatal (preterm birth, low birth weight, small for gestational age, and death) and postnatal stages (growth indicators, micronutrient deficiencies, and infant mortality) up to 6 months of age in LMICs. As indicators of maternal nutritional status, we specifically searched for (i) maternal anthropometry [gestational weight gain (GWG), weight, height, mid-upper arm circumference (MUAC), and body mass index (BMI)]; (ii) macro or micronutrient concentrations or deficiencies including nutritional anemia; and (iii) dietary intake of macronutrients/supplemental nutrition (protein/energy, iron, micronutrients and combinations) as shown in the conceptual framework.

## 2. Materials and Methods

### 2.1. Search Strategy

The protocol for this overview of reviews was registered with PROSPERO: CRD42024555199 (the international prospective register of systematic reviews, www.crd.york.ac.uk/prospero/, last accessed on 17 October 2024) and is reported following the PRISMA guidelines. The preliminary search of core electronic databases for health sciences Medline was performed through Ovid (PubMed) and the Cochrane Database of Systematic These databases were prioritized in our search due to resource constraints and because they index most systematic reviews [18]. Reviews started on July 2023, with verification and extended keywords added on 26 June 2024. Databases were searched from 2013 to the present to capture reviews conducted in the last 10 years since the 2013 Lancet Maternal and Child Nutrition Series for an up-to-date overview of the evidence. A validated search filter for reviews in PubMed was used [19]. References included studies that were hand-searched to identify additional reviews for potential inclusion. See the Supplementary Files Table S1 for a detailed overview of search terms.

### 2.2. Search Selection

Any review studies conducted in the past 10 years (2013 to 2023), including studies from low-and middle-income countries, as defined by the 2023 World Bank classification [20], and reporting on maternal nutritional status indicators and associated fetal or newborn or infant morbidity or mortality outcomes, were eligible for inclusion. Table 1 describes detailed eligibility criteria. We excluded reviews if they contained only HICs evidence, if maternal nutritional status was related only to maternal obesity, and if they did not report on outcomes in offspring. Overviews of reviews were included to search for additional reviews of relevance. Two reviewers independently screened the studies and resolved any disagreements through discussion. Expert opinion was sought on the reference list of reviews included in this overview.

### 2.3. Data Synthesis

Pooled effect estimates from reviews were extracted and reported as-is. We extracted information on the setting where primary studies were conducted to generate an LMIC-specific effect estimate using Revman 5.4 by following methods described in source reviews. If meta-analysis was not possible due to a limited number of studies, single-study effect estimates were reported, or effects were narratively summarized. To summarize the information from narrative reviews, studies reporting maternal factors associated with undernutrition indicators in children were tallied. Data from reviews (type and year of included studies, setting of studies, relevant maternal factors, associated outcomes in offspring, and strength of evidence) were extracted by the first reviewer and verified by the second reviewer. An evidence-based decision tool was used to guide decisions on overlapping reviews, and all reviews of relevance were prioritized for data extraction [21]. A citation matrix was created for each outcome under each comparison, listing all systematic reviews alongside their associated primary studies. To avoid double counting outcome data from overlapping reviews, the second reviewer ensured that each primary study’s outcome data were extracted only once if reported in multiple reviews. The methodological quality of the included systematic reviews was assessed using the Measurement Tool to Assess Systematic Reviews-2 (AMSTAR-2) instrument [22] and graded as (1) very low, (2) low, (3) moderate, and (4) high-quality reviews. For non-systematic reviews, we verified that the quality assessments of primary studies were reported. Systematic reviews with an AMSTAR-2 score below four were excluded at the extraction phase due to critical flaws that undermine their reliability, as indicated by the tool’s guidance [21,22].

## 3. Results

**Figure 2:**
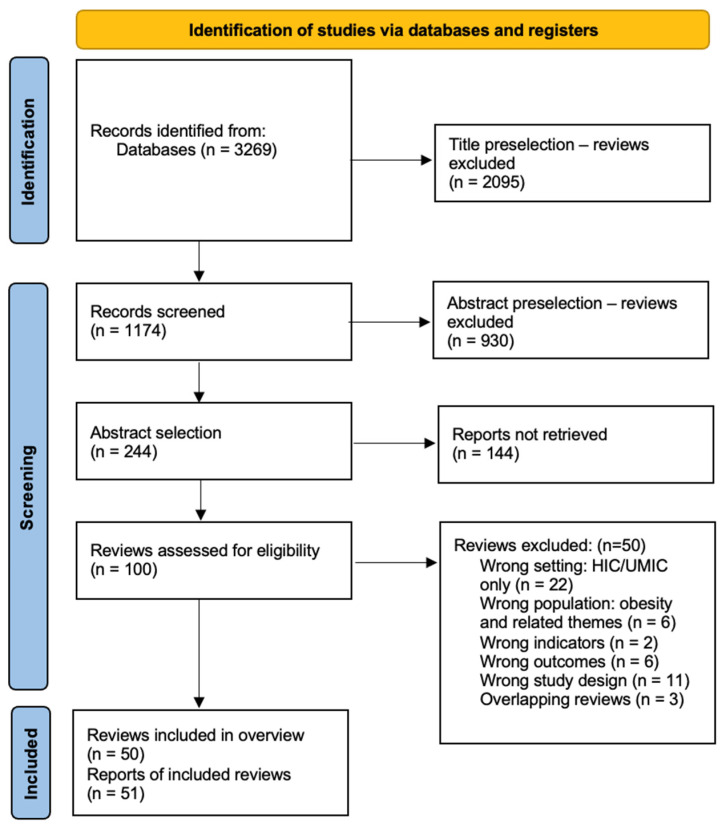
PRISMA Flow Diagram.

The initial search, in 2023, yielded 2624 results, of which 38 reviews of relevance were found after full-text screening. The updated search in 2024 (645 results) contributed an additional 64 new full-text reviews, from which 12 were selected. In total, this overview included 50 reviews. See Figure 2 for the PRISMA flowchart. A list of excluded studies and the PRISMA checklist can be found in Supplementary Files Tables S2 and S4, respectively.

Reviews were grouped according to maternal nutritional indicators/supplementation during (1) the peri-conception period and first trimester (*n* = 5 reviews), (2) early pregnancy (first and second trimesters or <20 weeks’ gestation) (*n* = 17 reviews), (3) late pregnancy (third trimester or delivery time) (*n* = 10 reviews), (4) post-partum and lactation periods outcomes (*n* = 3 reviews), and their associations with fetal, neonatal and infant outcomes, as shown in Table 2, Table 3, Table 4 and Table 5.

Given that 21 of the included studies did not report data by trimester, studies were also grouped according to maternal factors studied in association with fetal/neonatal and infant outcomes as (1) maternal diet (*n* = 3 reviews), (2) maternal anthropometry (*n* = 8 reviews), (3) maternal hemoglobin (Hb)/anemia (*n* = 5 reviews), (4) maternal micronutrient status (*n* = 6 reviews), (5) maternal supplementation with individual or combined macro/micronutrients (*n* = 28 reviews), detailed in Supplementary Files Table S3.

This overview included 13 reviews from the Cochrane group, all on maternal supplementation [23,24,25,26,27,28,29,30,31,32,33,34,35]. Although initially excluded by our protocol, seven narrative reviews were also included since they reported tallied associations [36,37,38,39,40,41,42,43]. The included reviews comprised the following types of included studies: quantitative intervention (RCT, case-control) and observational (cohort, cross-sectional) studies.

**Figure 3:**
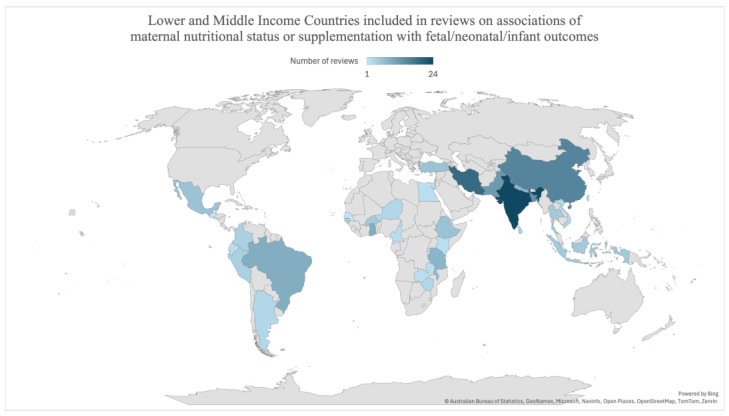
Map showing the distribution and number of low- and middle-income countries included in reviews on associations of maternal nutritional status or supplementation with fetal, neonatal, and infant outcomes.

Most selected reviews included both HICs and LMICs. Figure 3 shows the number of times LMICs were included in reviews on associations of maternal nutritional status or supplementation with fetal, neonatal, or infant outcomes.

### 3.1. Associations Between Maternal Nutritional Indicators or Supplementation and Fetal, Neonatal, and Infant Outcomes by Time of Pregnancy

#### 3.1.1. Periconception Period

Reviews studying the periconceptional period included studies mostly from Southeast Asia, with fewer studies coming from African, Eastern Mediterranean, Western Pacific, and Latin American regions. Reviews described the impact of maternal diet, nutritional status, and folate supplementation.

##### Maternal Diet

Antenatal nutritional education intending to increase energy and protein intake showed an effect on increasing birthweight among undernourished women and decreased the risk of LBW [44]. Prenatal small-quantity (SQ)lipid-based nutrient supplementation (LBS) showed a reduced risk of LBW in Ghana but no impact in Malawi [40]. Reviews not specifying the time of pregnancy found weak evidence that a healthy dietary pattern was associated with greater weight gain, but no association was found with the odds of inadequate or excessive GWG [45]. Also, the intake of <5/10 food groups during 24 h recalls (based on Food Frequency Questionnaires) (FFQ) was associated with increased odds of LBW [46].

##### Maternal Anthropometry

Prepregnancy underweight was also found to be associated with an increased risk of preterm birth, small for gestational age [47,48], and with LBW in studies conducted in Southeast Asia and the Eastern Mediterranean regions (Table 2).

On the other hand, four studies from Sub-Saharan Africa reported a higher proportion of LBW in babies from women with low GWG, but estimates were not provided [49]. A more recent review found strong evidence that severely and moderately inadequate GWG was associated with LBW in LMICs, but evidence was not conclusive for other adverse outcomes (PTB, stillbirth, or neonatal death) [41]. Others found that higher maternal BMI and GWG had positive associations with birth weight [13]. In contrast, maternal low BMI [42,50], poor GWG, anemia, and hypoproteinemia were associated with IUGR [50]. Despite these associations, maternal anthropometry showed not sufficient sensitivity or specificity for the prediction of SGA [51].

##### Maternal Folate Supplementation

Daly et al. [52] confirmed the high-grade evidence that periconceptional folate decreases the odds of neural tube defects (NTD) [53].

**Table 2:** Preconception or First Trimester.

Maternal Nutritional Status Indicator	Reviewed by	Quality of the Review	Outcome	Overall			LMICs	
				Effect Estimate (95% CI)	Number of Studies (Participants)	Quality of Evidence	Effect Estimate (95% CI)	Number of Studies (Participants)
Antenatal nutritional education	Ota et al. (2015) [29]	High	BW among under-nourished women	MD: 489.76 (427.93 to 551.59,	2 (320)	Low ^1^	MD: 490 (427.40 to 552.60)	1 (300) (Bangladesh)
			LBW	RR: 0.04 (0.01 to 0.14)	1 (300)	Low ^1^	LMIC evidence only (Bangladesh)	
Multivitamins + IFA vs. IFA	Balogun, O. O., et al. (2016) [33]	High	Stillbirth	RR 0.92, (0.85 to 0.99)	10 (79,851)	High ^1^	LMIC evidence only (Pakistan, Tanzania, Nepal, Burkina Faso, Niger (1 each) Indonesia (3), Bangladesh (2))	
			Early or late miscarriage	RR 0.98, (0.94 to 1.03)	10 (94,948)	Moderate ^1^	LMIC evidence only (Pakistan, Tanzania, Nepal, Burkina Faso, Niger (1 each) Indonesia (3), Bangladesh (2))	
Folate supplementation	Daly, M., et al. (2022) [52]	High	Neural tube defects	RR: 0.31 (0.17 to 0.58)	5 (6708)	High ^1^	RR: 0.41 (0.19 to 1.29)	1 (279) (India)
Prepregnancy underweight	Rahman et al. (2015) [48]	Low	PTB	OR: 1.13 (1.01 to 1.27)	11	Moderate-High ^2^	LMIC evidence only (China (4), Iran (2), Argentina, Thailand, Pakistan, Mexico, Thailand (1 each))	
	Dean et al. (2014) [47]	Low		OR: 1.32 (1.22 to 1.43)	12	Low ^1^	OR: 0.90 (0.40 to 2.02)	1 (China)
	Rahman et al. (2015) [48]	Low	SGA	OR: 1.85 (1.69 to 2.02)	5	Moderate-High ^2^	LMIC evidence only (China (3), Brazil (2))	
	Dean et al. (2014) [47]	Low		RR: 1.64 (1.22 to 2.21)	4	Low ^1^	OR: 1.95 (1.52 to 2.50)	1 (Vietnam)
	Rahman et al. (2015) [48]	Low	LBW	OR: 1.66 (1.50 to 1.84)	8	Moderate-High ^2^	LMIC evidence only (China (3), Thailand (2), Pakistan, Mexico, Iran)	
	Dean et al. (2014) [47]	Low		RR: 1.37 (0.46 to 4.13)	5	Low ^1^	RR: 0.74 [0.39, 1.43] OR:1.97 [1.18, 3.28] (Han 2010 [54]; referent BMI 18.5–22.9 for Asian population)	2 (China and Vietnam)

^1^: GRADE Assessment, ^2^: Newcastle–Ottawa instrument.

#### 3.1.2. Early Pregnancy

Africa, Latin America, and Southeast Asia were represented in this group of studies, with the inclusion of only one country for the Western Pacific (China) and Eastern Mediterranean regions (Iran). Reviews studying maternal factors during early pregnancy focused on maternal specific nutritional deficiencies and supplementation (Table 3).

##### Maternal Diet

One review on dietary intakes during early pregnancy included three studies from LMICs [55], showing that an increased frequency or intake of fruits and vegetables starting in the second trimester was associated with increased birth weight, as found in one study from Egypt [56], but no association was found in two other studies from India [57,58].

**Table 3:** Early pregnancy.

Maternal Nutritional Status Indicator	Reviewed by	Quality of the Review	Outcome	Overall			LMICs	
				Effect Estimate (95% CI)	Number of Studies (Participants)	Quality of Evidence	Effect Estimate (95% CI)	Number of Studies (Participants)
Fruit and vegetable intake in the second or third trimester	Murphy, M. M., et al. (2014) [55]	Very Low	BW	One study reported positive association	4 (1214) (India (2), Malaysia, Egypt)	Very low ^1^	Increased frequency of intake in second and third trimester associated with higher BW	1 (234) (Egypt)
Anemia in the first trimester	Rahmati et al. (2017) [59]	Very low ^3^	LBW in the first trimester of pregnancy	OR: 1.26 (1.03 to 1.55)	12 (210,578)	Low ^4^	OR: 0.96 (0.85 to 1.08)	10 (34,383)
Low Hb in the second trimester	Dewey, K. G. and B. M. Oaks (2017) [37]	Not a systematic review	PTB	Three studies report positive association	11 (374,925)	Low ^1^	1/6 studies in LMICs positive association	1 (35,449) (Peru)
			SGA	Three studies report positive association	10 (214,252)	Low ^1^	2/6 studies in LMICs positive association	2 (36,872) (Peru and Malawi)
			Stillbirth	Two studies report positive association	5 (428,091)	Low ^1^	1/6 studies in LMICs positive association	1 (35,449) (Peru)
Maternal B12 deficiency	Sukumar, N., et al. (2016) [60]	Very Low	LBW/SGA	OR: 1.70 (1.16 to 2.50)	8 (1482)	Moderate ^1^	OR: 2.44 (1.50 to 3.95)	6 (1032) (India)
			Vitamin B12 concentrations in maternal blood	MD: −9.12 (−21.25, 3.01)	14 (1969)	Low ^1^	A larger effect size found in the first and second compared with the third trimester.	4 (India (3), Pakistan))
Iron <20 weeks’ gestation	Cantor, A. G., et al. (2015). [61]	Low	SGA	Inconsistent effect	4 (2595)	Fair-Good ^5^	SGA in women who received supplements: 15% vs. control: 10% [*p* = 0.035])	1 (727) (Iran)
Iron treatment started <20 weeks’ gestation vs. placebo	Peña-Rosas, J. P., et al. (2015) [30]	High	LBW	RR: 0.79 (0.59 to 1.05)	6 (14,512)	Low ^1^	RR: 0.73 (0.53 to 1.00)	4 (13,965) (China, Nepal, Iran (2))
Serum 25 (OH)D levels <75 nmol/L	Amegah, A. K., et al. (2017) [62]	Very low	PTB <35–37 weeks	RR: 1.13 (0.94 to 1.36)	7	Moderate ^1^	RR: 1.04 (1.02 to 1.06)	1 (China)
Vitamin D supplementation <20 weeks’ gestation			APGAR score	Vitamin D nutrition status was positively correlated with APGAR scores	2	Very Low ^1^	LMIC evidence only (India, Pakistan)	
Vitamin D supplementation <20 weeks’ gestation,	Zhao, R., et al. (2022) [63]	Very Low	LBW	RR: 0.65 (0.48 to 0.86)	14	Low ^1^	RR: 0.60 (0.41 to 0.89)	9
Ca supplementation starting around week 20	Hofmeyr, G. J., et al. (2018) [26]	High	PTB	RR: 0.76 (0.60 to 0.97)	11 (15,275)	Low ^1^	RR: 0.68 (0.49 to 0.95	5 (2099) (Argentina, Ecuador (2), India (2))
Zinc supplementation <27 weeks’ gestation	Carducci, B., E. C. Keats and Z. A. Bhutta (2021) [23]	High	PTB	RR: 0.87 (0.74 to1.03)	21 (9851)	Low ^1^	RR 0.98 (0.87 to 1.11)	13 (5724)
			Stillbirth	RR: 1.22 (0.80 to 1.88)	7 (3295)	Low ^1^	RR 1.34 (0.85 to 2.12)	5 (2310)
			LBW	RR: 0.94 (0.79 to 1.13)	17 (7399)	Moderate ^1^	RR 1.05 (0.96 to 1.15)	11 (4957)
			SGA	RR: 1.02 (0.92 to 1.12)	9 (5330)	Moderate ^1^	RR 1.05 (0.97 to 1.13)	5 (2330)
Omega-3 supplementation <21 weeks’ gestation,	Saccone, G., et al. (2015) [64]	Low	Perinatal mortality	Overall: RR: 0.61 (0.30 to 1.24) <21 weeks: RR: 0.27 (0.09 to 0.80)	5 (3415)	Low ^1^	RR: 1.13 (0.51 to 2.49)	1 (323) (Bangladesh)
Antioxidant levels	Solé-Navais, P., et al. (2016) [43]	Not a systematic review	BW	Inverse association of elevated homocysteine mid-pregnancy with BW	3 (1514)	Low ^1^	Non-significant association in one study	2 (1041) (India (2))
MMN <20 weeks’ gestation vs. IFA supplementation	Bourassa, M. W., et al. (2019) [39]	High	PTB	RR: 0.93 (0.87 to 1.00)	14	Moderate ^1^	LMIC evidence only (Nepal (1), Tanzania (1), Guinea Bissau (1), China (1), Nepal (1), Mexico (1), Burkina Faso (1), Bangladesh (2))	
LBS <20 weeks’ gestation vs. IFA supplementation	Das, JK., et al. (2018) [25]	High	LBW	RR 0.87 (0.72 to 1.05)	3 (4826)	Moderate ^1^	LMIC evidence only (Bangladesh, Ghana, Malawi)	
			SGA	RR 0.94 (0.89 to 0.99)	3 (4823)	Moderate ^1^		
			PTB	RR 0.94 (0.89 to 0.99)	3 (5924)	Moderate ^1^		
Food fortification at <20 weeks’ gestation,	Gresham, E., et al. (2014) [65]	Low	Birth weight, low birth weight, length, head circumference	Lower incidence of LBW infants	1 (1135)	Neutral ^6^	LMIC evidence only (Chile)	
High-energy supplement			Neonatal birth and weight, birth and weight up to 1 year of age	Heavier and taller infants at 3, 6 and 12 months of age	1 (542)	Neutral ^6^	LMIC evidence only (Indonesia)	
Fortified biscuits			Stillbirth, birth weight, low birth weight, length, and head circumference	Reduced prevalence of LBW. stillbirths and larger head circumference	1 (2082)	Positive ^6^	LMIC evidence only (Gambia)	

^1^: GRADE Assessment, ^3^: Information from the narrative, as sensitivity analyses by trimester were not conducted, ^4^: STROBE checklist and publication bias assessment, ^5^: USPSTF quality assessment tool, ^6^: American Dietetic Association Quality Criteria Checklist for Primary Research.

##### Maternal Micronutrient Deficiencies

Maternal anemia during the first or second trimesters has been shown to increase the risk of LBW, PTB, perinatal mortality, and neonatal mortality [66]. However, Dewey et al. found that both low and high hemoglobin (Hb) concentrations during the second trimester were associated with an increased risk of PTB, SGA, and stillbirth, and that high Hb in the third trimester increased the risk of LBW [37]. Vitamin B12 deficiency has also been associated with LBW and SGA, with a larger effect size during the first and second trimesters [60].

##### Maternal Supplementation

Regarding the effect of supplementation during early pregnancy and offspring outcomes, individual micronutrient supplementation during the second trimester has shown positive impacts on the offspring. Women receiving iron supplementation before 20 weeks’ gestation had a lower proportion of SGA newborns [61] and decreased the risk of LBW [30]. Those findings are not supported by other reviews not distinguishing between specific times of supplementation, where no difference in birth outcomes with iron supplementation during pregnancy was observed [67]. Vitamin D supplementation starting before 20 weeks’ gestation was associated with a reduced risk of LBW [63] and with improved Apgar scores [62], whereas calcium supplementation starting around 20 weeks’ gestation has been shown to decrease the risk of PTB [26].

On the other hand, no difference was found between women taking or not taking zinc supplements before 27 weeks’ gestation for the risk of PTB, LBW, and SGA [23]. Although two reviews showed that the intake of omega-3 fatty acid supplementation starting during the first half of pregnancy was associated with lower perinatal death in high-income countries (HIC), subgroup analyses did not include studies from LMICs [64,68].

Supplementation with multiple micronutrients (MMN) plus iron folic acid (IFA) starting before 20 weeks’ gestation was associated with decreased odds of stillbirth [33] and reduced the risk of PTB [39], but the effect on PTB was not observed when supplementation started after 20 weeks’ gestation [39].

Macronutrient supplementation approaches, with or without micronutrients during the second trimester, have been shown to help improve neonatal outcomes. For example, LBS starting before 20 weeks’ gestation was associated with higher weight and length at birth and a decreased risk of SGA when compared with IFA [25]. As reviewed by Gresham et al., early in-pregnancy initiation of milk-based supplementation improved BW and decreased LBW in Chilean mothers, a high-energy supplement increased weight and length at birth to year, compared with low-energy supplements in Indonesia, and fortified biscuits reduced LBW and stillbirth and increased head circumference in Gambia [65].

#### 3.1.3. Late Pregnancy

Like early pregnancy, reviews studying maternal factors during late pregnancy had a similar country representation, and also focused on maternal specific nutritional deficiencies and supplementation (Table 4).

##### Maternal Micronutrient Deficiencies

Whereas Figueiredo et al. [69] did not find an association between maternal anemia and LBW after sensitivity analyses by trimester, Dewey et al. [37] reported that low Hb in the third trimester was associated with an increased risk of stillbirth and PTB. Moreover, they also reported one study showing that the risk of stillbirth was increased by both low and high Hb during the third trimester [70]. Reviews not reporting sensitivity analyses by trimester found that maternal anemia increased the odds of LBW [13,71], PTB, and perinatal mortality [71].

**Table 4:** Late pregnancy.

Maternal Nutritional Status Indicator	Reviewed by	Quality of the Review	Outcome	Overall			LMICs	
				Effect Estimate (95% CI)	Number of Studies (Participants)	Quality of Evidence	Effect Estimate (95% CI)	Number of Studies (Participants)
Maternal anemia	Figuerido et al. (2018) [69]	Moderate	LBW	Overall— Adjusted OR: 1.23 (1.06 to 1.43) Third trimester— Crude OR: 0.88 (0.53 to 1.48)	Overall—13 third trimester -3	Moderate-High ^2^	OR: 1.30 (0.87 to 1.94)	4
Low hemoglobin in the third trimester	Dewey et al. (2017) [37]	Not a systematic review	Stillbirth	Two studies report negative association	3 (190,849)	Low ^1^	Two studies report negative association	2 (168,050) (Iran and China)
Iron supplementation at >20 weeks’ gestation	Peña-Rosas, J. P., et al. (2015) [30]	High	LBW	RR: 1.05 (0.50 to 2.19)	3 (665)	Low ^1^	RR: 0.57 (0.14 to 2.31)	1 (181) (Gambia)
Low maternal or cord blood B12	Sukumar, N., et al. (2016) [60]	Very Low	LBW/SGA	OR: 1.70 (1.16 to 2.50)	8 (1482)	Moderate ^1^	OR: 2.44 (1.50 to 3.95)	6 (1032) (India)
Plasma folate/B12 in the third trimester	Solé-Navais, P., et al. (2016) [43]	Not a systematic review	Infant growth outcomes	Plasma homocysteine negatively associated with BW.	3 (1514)	Low ^1^	Non-significant association in one study	2 (1041) (India (2))
Maternal vitamin D in the second and third trimesters	Dos Santos et al. (2023) [72]	Not a systematic review	PTB	RR: 7.35 (2.99 to 18.07)	1 (180)	Very Low ^1^	LMIC evidence only (Brazil)	
Vitamin D supplementation >20 weeks’ gestation	Zhao, R., et al. (2022) [63]	Very Low	PTB	RR: 0.67 (0.57 to 0.79)	53	Low ^1^	RR: 0.62 (0.46 to 0.84)	22
Calcium supplementation >20 weeks’ gestation (no supplementation <20 weeks)	Buppasiri et al. (2015) [24]	High	PTB	RR: 0.86 (0.70 to 2.05)	13 (161,390)	Moderate ^1^	RR: 0.92 (0.82 to 1.04)	6 (10,622) (Argentina, Iran, multicenter in LMICs, India (3))
			LBW	Overall: RR: 0.93 (0.81 to 1.07) After 20 weeks RR 0.41 (0.23 to 0.73)	Overall: 6 (14,162) After 20 weeks: 3 (737)	Moderate ^1^	0.99 (0.93 to 1.05)	3 (8928) (Argentina, Ecuador, India)
MMN >20 weeks’ gestation	Bourassa et al. (2019) [39]	High	SGA	RR: 0.94 (0.90 to 0.98) Third trimester RR: 0.88 (0.79 to 0.98)	Overall: 17 third trimester: 6	Moderate ^1^	LMIC evidence only Pakistan (1), Nepal (1), Ghana (1), Zimbabwe (1), Burkina Faso (1), Indonesia (1), Bangladesh (1))	
MNS >20 weeks’ gestation,	Haider, B. A. and Z. A. Bhutta (2017) [34]	Not a systematic review	Perinatal mortality	RR: 1.01 (0.91 to 1.13)	12	High ^1^	LMIC evidence only (Pakistan, Nepal (2), Tanzania, Zimbabwe, Guinea, Bissau, China (2), Mexico, Burkina Faso, Thailand (3), Bangladesh, Niger)	
BEP supplementation	Ota et al. (2015) [29]	High	Stillbirth	RR: 0.6 (0.39 to 0.94)	5 (3408)	Moderate ^1^	RR: 0.52 (0.31 to 0.88)	4 (2862) (Gambia, India, Burkina-Faso, Colombia)
			SGA	RR: 0.79 (0.69 to 0.90)	7 (4409)	Moderate ^1^	RR: 0.57 (0.50 to 0.66)	4 (2344) (Burkina-Faso, Colombia, Gambia, India)
			BW	MD: +41g (4.7 to 77.3 g)	11 (5385)	Moderate ^1^	MD: +59.71 (25.60 to 93.82)	5 (2228) (Gambia, India, Burkina Faso, Indonesia, Colombia)
MMN supplementation + enriched food during the third trimester	Gresham, E., et al. (2014) [65]	Low	BW	Women produced heavier full-term male infants [95 g (*p* < 0.05)] compared to control	1 (456)	Neutral ^3^	LMIC evidence only (Colombia)	

^1^: GRADE Assessment, ^2^: Newcastle–Ottawa instrument, ^3^: Information from the narrative, as sensitivity analyses by trimester were not conducted.

Maternal vitamin B12 deficiency during the third trimester was found to be associated with LBW and SGA [60]. Also, low B12/folate concentrations during the third trimester were associated with lower birth weight, length, and head circumference while increasing the odds of PTB [43]. The review from Solé–Navais et al. reported a negative association between plasma homocysteine and birth weight [43]. These findings are supported by another review not performing sensitivity analyses by trimester, where higher maternal B12 concentrations were associated with a reduced risk of PTB, and B12 deficiency was associated with an increased risk of LBW [73]. Counterintuitive findings were reported by Dos Santos et al. [72], in which one of the studies from Brazil found that higher maternal vitamin D during the three trimesters was associated with an increased risk of preterm birth [first trimester incidence rate ratios (IRR) = 1.02; 95% CI 1.002; 1.03; *p* = 0.03; second trimester IRR = 1.05, 95% CI 1.03; 1.07, *p* < 0.001; third trimester IRR = 1.04, 95% CI 1.02; 1.06, *p* < 0.001] [74]. Also, one study from India, in the review from van der Pligt [75], found an association between vitamin D deficiency (trimester not specified) and LBW.

##### Maternal Supplementation

Regarding micronutrient supplementation, when provided after 20 weeks’ gestation, vitamin D supplementation reduced the risk of PTB and SGA [63], but a review from Palacios et al. [35] found no effect of vitamin D supplementation before or after 20 weeks’ gestation on the risk of PTB. Calcium supplementation reduced the risk of LBW [24], whereas antioxidant supplements, vitamins C and E, have shown no effect on fetal, perinatal, or neonatal death, PTB, or IUGR, independently of the time of supplementation. Similarly, maternal zinc supplementation during pregnancy, independent of its duration, had no effect on birthweight or LBW [76].

MMN supplementation starting after 20 weeks’ gestation was shown to reduce the risk of perinatal mortality [34] and led to a better reduction in the risk of stillbirth, compared with supplementation <20 weeks’ gestation [39]. The review from Haider et al. also showed that MMN supplementation reduced the risk of PTB in women with low BMI and a reduced risk of SGA in women taller than 154.9 cm, without specification of trimester [34]. Without sub-analyses by the time of supplementation, Bhutta and Das [77] reported that iron/IFA supplementation was associated with a higher birthweight and reduced risk of LBW. They also reported that calcium supplementation during pregnancy was associated with a higher birthweight and decreased risk of PTB and that MMN supplementation decreased the risk of LBW and SGA.

Balanced protein energy (BPE) supplementation during the third trimester increased birthweight, reduced the risk of stillbirth and SGA, and increased birthweight [29]. Gresham et al. [65] included in their review one study using a combination of dry skim milk, protein-enriched bread, vegetable oil, and vitamin-mineral supplementation during the third trimester, which was shown to improve birthweight [78].

Other reviews not performing analyses by trimester showed that BEP supplementation was associated with increased GWG [13] and birthweight [13,41,77,79,80], but both positive [80] or no association [79] have been found for birth length, and head circumference. BEP has also been found to decrease the risk of SGA, stillbirths/perinatal mortality [13,41,77,80], LBW, and infant mortality [80].

#### 3.1.4. Post-Partum or Lactation

Reviews included mostly African populations, with a lower representation of studies from Southeast Asia (India) and Latin America (Brazil). Only three reviews described associations of maternal nutritional status/supplementation during the postpartum or lactation periods with offspring outcomes (Table 5).

Akombi et al. [36] showed that in African countries, low maternal BMI (8 studies) was associated with infant underweight, stunting, and wasting. This review also found that the intake of low-energy-density foods (1 study), low intake of fruits and vegetables (1 study), and low maternal height (1 study) were associated with infant stunting.

**Table 5:** Post-partum or lactation.

Maternal Nutritional Status Indicator	Reviewed by	Quality of the Review	Outcome	Overall			LMICs	
				Effect Estimate (95% CI)	Number of Studies (Participants)	Quality of Evidence	Effect Estimate (95% CI)	Number of Studies (Participants)
Low maternal BMI	Akombi et al. (2017) [36]	Not a systematic review	Wasting	Narrative	3 (36,223)	Medium ^4^	LMIC evidence only (Nigeria, Ghana, Ethiopia)	
			Stunting	Narrative	5 (41,070)	Medium-high ^4^	LMIC evidence only (Ghana (2), Nigeria, Ethiopia, Tanzania)	
Intake of low-energy density foods			Stunting	Narrative	1 (261)	Medium ^4^	LMIC evidence only (Uganda)	
Low intake of fruits and vegetables			Stunting, wasting, underweight	Narrative	1 (1963)	Medium ^4^	LMIC evidence only (Cameroon)	
Low maternal height			Stunting	Narrative	1 (318)	Medium ^4^	LMIC evidence only (Ethiopia)	
Breastmilk micronutrients	Reyes et al. (2024) [38]	High	Infant growth outcomes	No association: breastmilk Vit. A, B, K and Mg, Cu, Fe, and infant anthropometry: Mixed results: breastmilk Ca, Na, P, Zn, I, Se, and infant anthropometry. Positive association: Mg in breastmilk with higher WAZ	26 (2526)	Good ^5^	Most studied mineral in LMICs Zinc: 8 studies	17 (2298)
Maternal vitamin A post-partum supplementation	Oliveira et al. (2016) [28]	High	Infant mortality	RR: 1.08 (0.77 to 1.52)	5 (6090)	Low ^1^	LMIC evidence only (Kenya, India, Ghana, Zimbabwe, Brazil)	
			Neonatal morbidity (gastroenteritis at 3 months)	RR: 6.03 (0.30 to 121.82)	1 (84)	Very low ^1^	LMIC evidence only (India)	

^1^: GRADE Assessment, ^4^: STROBE checklist and publication bias assessment, ^5^: USPSTF quality assessment tool.

One review studied the impact of multiple breastmilk constitutive nutrients on infant anthropometry, showing that most studies measuring breastmilk zinc and magnesium found positive associations of these micronutrients with infant anthropometry, whereas mixed results were found for calcium, and no association was found for the content of iron in breastmilk with infant anthropometry [38].

Maternal vitamin A supplementation, although improving breastmilk vitamin A concentrations, showed no effect on infant mortality or morbidity [28], and any duration of vitamin A supplementation during pregnancy showed no effect on perinatal/neonatal mortality or stillbirth [27].

## 4. Discussion

This overview or review highlighted limited information on associations between maternal nutritional status or supplementation in the preconception period, early and late pregnancy, and during postpartum or lactation with offspring outcomes in LMICs. Results on associations varied when analyses were run by trimester.

### 4.1. Key Messages of Associations of Maternal Nutritional Status at Different Stages

(1) Around conception, a low intake of fruits and vegetables, as well as low GWG and underweight, were risk factors for adverse fetal (miscarriage and IUGR) and neonatal (PTB, SGA, and LBW) outcomes. (2) During early pregnancy, whereas the impact of fruits and vegetables showed mixed results, stronger evidence was found on the impact of maternal anemia or high Hb and low vitamin B12 status on adverse neonatal outcomes (PTB, LBW, SGA, stillbirth, neonatal mortality). (3) During late pregnancy, findings on the effect of maternal anemia are not consistent, demonstrating either no effect on adverse neonatal outcomes or an increased risk of PTB and SGA, but findings suggest that both anemia and high Hb concentrations during the third trimester can increase the risk of stillbirth. Low maternal B12/folate status during early pregnancy has shown consistent associations with an increased risk of PTB, LBW, and SGA. On the other hand, although associations of vitamin D deficiency with PTB and LBW have been reported, there is limited evidence from LMICs. (4) During post-partum/lactation, maternal inappropriate diet and undernutrition have shown associations with infant underweight, stunting, and wasting, and there is evidence suggesting that the micronutrient content of breastmilk, particularly zinc and magnesium, may have an impact on infant anthropometry. Reviews on supplementation did not always report the time during pregnancy when administered, but our findings suggest that improving maternal nutritional status at the beginning of pregnancy may have a higher impact on neonatal outcomes, in agreement with crucial roles of individual macro and micronutrients at different stages of fetal development [81]. Current evidence points to MMN or BPE/LBS supplementation to have more benefit than supplementation with individual micronutrients. Even if there is some evidence of the beneficial effects of fruits and green leafy vegetable intake during pregnancy for improving birthweight [46,55], there is a gap in research studying dietary intakes during pregnancy and adverse outcomes, specifically targeting important groups of macro and micronutrients. Diets with potential proinflammatory properties have been associated with SGA, LBW, and with large for gestational age and obesity later in life in developed settings [82]. Also, healthy dietary patterns such as the alternate Mediterranean diet and dietary approaches to stop hypertension are able to improve neonatal outcomes such as birth weight, birth length [83], and PTB [84] in the US, but studies in LMICs are lacking.

Reviews in our search showed that low BMI during prepregnancy and pregnancy [47], low GWG [51], and maternal undernutrition [42,48,50] were important determinants of adverse fetal outcomes, notably SGA and IUGR. Maternal height was studied as an independent factor associated with LBW by only one review [13], where the important increased risk (52%) makes a case for other studies to explore this association. Also, only one review [36] narratively explored associations of maternal nutritional status with children beyond the perinatal period, suggesting that the effect of maternal undernutrition can extend to preschool age, given associations of low maternal weight and height with child stunting and of low maternal BMI with child wasting. These results align with recent reviews [14,16,85,86] showing that maternal indicators of undernutrition are independent determinants of child stunting and/or wasting, showing the important role of improving maternal general nutrition before and during pregnancy for the prevention of child undernutrition.

On the other hand, the theoretical benefit of individual micronutrients has not been fully evidenced in clinical trials. Studies from LMICs focused mostly on hemoglobin as a nutritional indicator, given its availability even in low-resource settings [87], but the study of micronutrients related to anemia is often limited due to inadequate funding to perform laboratory assays [88]. In general, studies used the term anemia interchangeably with iron deficiency, underscoring the multifactorial origin of anemia, which is often seen in deprived populations [89]. Other micronutrient deficiencies or inflammation were rarely reported, as these indicators are not part of large nutritional surveys but may have an impact when exploring associations between maternal anemia and infant outcomes. Despite these limitations, associations were observed between maternal Hb concentrations or anemia and offspring outcomes, which varied depending on the setting. Studies including only LMICs showed, in general, larger associations between maternal anemia and adverse pregnancy or infant outcomes [66,71]. Reported associations of low or high Hb with adverse pregnancy outcomes also varied depending on the stage of pregnancy, with supplementation being more effective when provided to anemic women at the beginning of pregnancy [37,59]. In this regard, it is important to recall that Hb measurement is affected by factors such as altitude and smoking, usually controlled for in epidemiological studies [90], but other factors such as plasma volume, which insufficient expansion during pregnancy is associated with adverse outcomes [91], have been rarely reviewed [92].

Systematic reviews on micronutrients other than iron were limited to vitamins B12 [43,60,73] and D [72,75], reporting evidence of their associations with adverse neonatal outcomes (SGA, LBW, and PTB) in individual studies, but no meta-analyses have been performed to determine pooled risk estimates. Observations of an increased risk of PTB with higher vitamin D concentrations [74] are of special concern. However, current research points to a protective role of vitamin D in PTB via the modulation of the inflammatory response that triggers the process of labor by improving antibacterial responses and by decreasing myometrial contractility [93]. The biological plausibility of vitamin D during pregnancy still needs to be confirmed by randomized clinical trials.

The lack of review studies on associations of maternal nutritional status during the lactation period with infant health was noticeable, but studies have shown lower anthropometry in infants from iodine insufficient mothers from China [94], improved infant anthropometry with maternal higher intake of animal source foods, intake of MNS and higher vitamin D concentrations in a cohort from Panama [95], and associations between maternal B12 status during lactation and child growth at 5 years in Nepal [96]. We found only one review showing associations between the micronutrient content of breastmilk and infant outcomes. The positive association of breastmilk calcium content with infant length found in Gambia [38,97] is worth further research.

### 4.2. Studies on Maternal Supplementation and Fetal/Newborn/Infant Outcomes

Iron supplementation had many variations in results depending mostly on the time of supplementation during pregnancy and the setting of the study. It has been observed that the benefit of iron supplementation varies from iron-deficient women to those who are iron-replete [37], explaining the variation in meta-analysis results depending on the prevalence of iron-deficient in their included studies [98]. Two earlier reviews showed associations between iron supplementation and a reduced risk of LBW [77] and SGA [61]. Others have shown conflicted effects on the increase in BW [13,30], and a later review found no effect of iron supplements on adverse perinatal outcomes while including only one study from LMICs [67]. Mechanisms of iron supplementation associations with adverse pregnancy outcomes include a possible increase of iron accessibility for oxidative stress and extracellular microbial utilization [98], as well as possible side effects of iron supplementation on increasing blood viscosity, disruption of intestinal microflora, and altered response to inflammation and infection [37,99]. Therefore, further research is needed in populations with high rates of infection/inflammation, notably in malaria-endemic areas, where the increased risk of maternal malaria with iron supplementation is currently not supported by epidemiological evidence [98]. On the other hand, observations that iron supplements could increase the risk of SGA/LBW [37] warrant caution in the selection of women for iron supplementation. To date, there is no conclusive evidence that maternal iron supplementation helps prevent adverse fetal or neonatal outcomes.

Whereas the role of folic acid supplementation for the prevention of neural tube defects has been reiterated [52], no role of supplementation with vitamin A (required for fetal growth, tissue maintenance and reserves, and for maternal metabolism) [27,28] or zinc (needed for appropriate embryogenesis and fetal development [100]) [23,76] has been found for improving pregnancy outcomes. Also, despite vitamins C and E have been studied together as antioxidants with a role in female reproductive and pregnancy pathologies [101], supplementation has failed to show any effect in decreasing the risk of adverse pregnancy outcomes [31] and the risk of premature rupture of membranes with vitamin E supplementation discourages its further use [31].

Given that a higher content of calcium in breastmilk may have a positive effect on the infant’s length and that calcium supplementation has been shown efficient for reducing PTB by two reviews [24,26], calcium supplements in deficient populations may have fetal benefits beyond their known effect in the reduction of hypertensive disorders of pregnancy [102]. On the other hand, despite the possible beneficial effects of vitamin D supplementation on perinatal outcomes, one Cochrane review found an increased risk of PTB with the combination of vitamin D and calcium supplementation [35]. This finding was based mostly on one study from Iran [103], which reported a lower mean duration of gestation in mothers supplemented with calcium + vitamin D [38.7 (38.5–38.9) weeks, *n* = 330] compared with aspirin treatment [39.0 (38.9–39.1), *n* = 330] and placebo [39.0 (38.9, 39.1), *n* = 330, *p* = 0.0001], and a higher (but non-significant, *p* = 0.16) prevalence of deliveries <37 weeks (13.7%) in women supplemented with Calcium + vitamin D, compared with those receiving aspirin (11.8%) and placebo (8.9%). This observation warrants caution when supplementing with both vitamin D and calcium but requires further studies focusing on settings with a high prevalence of both deficiencies while controlling for cofactors.

Studies on omega-3 fatty acid supplementation have been developed mostly in HICs, and those with the inclusion of LMICs in this review showed no conclusive effects on adverse perinatal outcomes [64,68]. A most recent review of studies in HICs has shown the beneficial effects of supplementation with omega-3 fatty acids during pregnancy for the reduction of LBW and PTB, and for improving newborn anthropometrics [104], but these results still need to be replicated in LMICs.

In line with reviews showing an improvement in fetal/neonatal outcomes with better nutritional status, BPE supplementation or lipid-based supplements, but not micronutrients alone or in combination, had a consistent effect on reducing the risk of stillbirth, LBW, SGA, and neonatal mortality [13,29,65,77,80]. In addition, lipid-based supplements showed an effect on improved newborn size [25,80]. Of note, included studies of BPE supplementation have been conducted mostly in African countries with a high prevalence of undernutrition, and results cannot be extrapolated to other settings. As recently reviewed by Shenoy et al. [105], many factors influence the effectiveness of nutritional interventions, including maternal education levels, the way interventions are communicated, and the degree of poverty and food insecurity. This review also found that the efficacy of individual micronutrient supplementation is surpassed when providing multiple micronutrient supplements in Asian settings [105]. Although we were limited by the information provided by the included reviews, our findings suggest that BPE/LBS, rather than individual micronutrient supplements, may better respond to nutritional needs during pregnancy in African LMICs.

There are several limitations to our study, mostly related to confounder factors that may help drive associations between maternal nutritional status and offspring outcomes. Most reviews included both HIC and LMIC, with a dearth of evidence for the latter, making it even more difficult to differentiate between low- and lower-medium-income countries. Also, not all reviews differentiate associations across the time of pregnancy or lactation and offspring outcomes, and maternal infection/inflammation status was not taken into account in all studies included in reviews. Lastly, the heterogeneity of review studies did not allow for subgroup analyses.

## 5. Conclusions

There is evidence that fetal, neonatal, and infant outcomes can be improved by interventions in mothers during pregnancy. Appropriate BMI and intake of fruits and vegetables during preconception and early pregnancy, adequate Hb concentrations, and associated micronutrients (iron, folate, vitamin B12) in mid and late gestation have proved to favor fetal, neonatal, and/or infant outcomes. Current evidence indicates that nutritional interventions during early pregnancy may be more effective in decreasing adverse offspring outcomes, and BPS/LBS may be more effective than supplementation with individual micronutrients, but policies and programs need to be adapted to the social and cultural contexts and particular nutritional needs of populations. In this regard, possible side effects of iron and antioxidant supplementation need further research in LMICs. Therefore, our research calls for caution when providing iron supplementation to populations with multiple nutritional deficiencies and infections. Finally, large studies investigating the long-term effect of maternal nutritional interventions while controlling for confounding factors (e.g., socio-demographic characteristics, infection/inflammation status, time of pregnancy) before pregnancy and after infancy are needed.

## Figures and Tables

**Table 1:** Criteria for the selection of studies.

Study Design Types	Population and Setting	Maternal Nutritional Indicator	Outcomes Associated in Offspring
**Inclusion criteria**			
Review studies conducted in the last 10 years (2013–2023)	Studies in low-and middle-income countries	Maternal nutritional status, indicated by: Anthropometry: gestational weight gain (GWG), weight, height, body mass index (BMI), mid-upper arm circumference (MUAC) Diet information, supplementation Macro/micro-nutrient measurements, anemia	**Fetal outcomes:** Intrauterine growth retardation (IUGR) Fetal death (>8 weeks term gestation) **Newborn outcomes:** Preterm birth (PTB) Low birth weight (LBW) Small for gestational age (SGA) Neonatal death (from birth to 28 days of age) **Infant outcomes:** Weight and length gain Growth indicators as continuous outcomes or their deficiency (<−2 SD): Weight for age Z-score (WAZ) or underweight Length/stature for age Z-score (LAZ) or stunting Weight for length Z-score (WAZ) or wasting. Micronutrient deficiencies (secondary outcome) Infant mortality (from birth to 1 year age)
**Exclusion criteria**			
Older reviews, animal studies, reviews focused on mechanistic pathways but not maternal offspring relationships, narrative reviews without pooled effects	Twin pregnancies, studies conducted in developed settings only	Maternal obesity and related themes (e.g., bariatric surgery) Non-nutritional anemias (sickle cell disease) Maternal comorbidities: endocrine disorders including thyroid diseases, gestational diabetes and diabetes, use of psychotropic substances, cholestasis, chronic diseases (e.g., chronic kidney disease), COVID-19	Child obesity Large for gestational age or macrosomia (LGA) as sole outcome Complications of VLBW or LBW Other specific neonatal outcomes (e.g., necrotizing enterocolitis, genetic syndromes)

## Data Availability

All published data is available in the public domain. Further inquiries can be directed to the corresponding author.

## Supplementary Materials

The following supporting information can be downloaded at https://www.mdpi.com/article/10.3390/nu16213725/s1: Table S1. Search strategy for targeted searches with additional terms; Table S2. Excluded reviews; Table S3. Characteristics of included reviews; Table S4. PRISMA Checklist.
