# Supplementary material for: Associations of Maternal Nutritional Status and Supplementation with Fetal, Newborn, and Infant Outcomes in Low-Income and Middle-Income Settings: An Overview of Reviews
Source: Nutrients. 2024 Oct 31;16(21):3725. doi: 10.3390/nu16213725 (PMC11547697; doi:10.3390/nu16213725)
Supplement: Supplementary file 1 [file nutrients-16-03725-s001.zip › nutrients-3275626-supplementary.pdf]

Supplementary Material - Associations of maternal nutritional status and supplementation with fetal, newborn, and infant outcomes in low-medium income settings: An overview of reviews

Doris González-Fernández [1], Oviya Muralidharan [1], Paulo A. Neves [1], Zulfiqar A. Bhutta [1]

1 Centre for Global Child Health, Hospital for Sick Children, Toronto, ON, M5G 0A4.

Table S1. Search strategy for targeted searches with additional terms

**Search strategy**	
Original search strategy for neonatal outcomes [2023]	(Pregnan* or preconcept* or pre-concept* or "pre concept*" or periconcept* or peri-concept* or "peri concept* " or gestat* or antenatal or "ante natal " or pre-natal or "pre natal " or prenatal or perinatal or peri-natal or "peri natal") and (maternal "muac" or "maternal height") and (iugr or "intra-uterine growth" or "intrauterine growth" or "intra uterine growth" or "gest* weight gain" or "fetal death" or "stillbirth" or “low birth weight” or “LBW” or “VLBW” or “small for gestational age” or “SGA”)
Original search strategy for infant outcomes [2023]	(“maternal” or “mother’s” or Pregnan* or preconcept* or pre-concept* or "pre concept*" or periconcept* or peri-concept* or "peri concept*" or gestat* or antenatal or "ante natal" or pre-natal or "pre natal" or prenatal or perinatal or peri-natal or "peri natal") and (BMI or "body mass index" or “maternal height” or “maternal weight” or “maternal MUAC”) or diet* or (nutr* or micronutr* or mineral* or vitamin* or deficien* or "anemia" or "anaemia") and (“infant weight” or “infant length” or “infant head” or “infant micronutrient*” or “infant anthropometry” or “underweight” or “stunting” or "low birth weight" or "Low-birth-weight" or "LBW" or "small for gestational age" or "small-for-gestational-age" or "SGA" or "very low birth weight" or "very-low-birth-weight" or "VLBW or “wasting” or “infant mortality”)
Updated search strategy [2024]	(“gestational weight gain” or “GWG”) and (iugr or "intra-uterine growth" or "intrauterine growth" or "intra uterine growth" or "gest* weight gain" or "fetal death" or "stillbirth" or “neonatal” or neonat* or “low birth weight” or “LBW” or “small for gestational age” or “SGA” or “infant weight” or “infant length” or “infant head” or “infant micronutrient*” or “infant anthropometry” or “underweight” or “stunting” or "low birth weight" or "Low-birth-weight" or "LBW" or "small for gestational age" or "small-for-gestational-age" or "SGA" or "very low birth weight" or "very-low-birth-weight" or "VLBW or “wasting” or “infant mortality”)
**Filters used**	
Study type – Review articles	("Systematic Review"[Publication Type:NoExp] OR "Systematic Reviews as Topic"[mesh:noexp] OR (("comprehensive"[TIAB] OR "integrated"[TIAB] OR "integrative"[TIAB] OR "mapping"[TIAB] OR "methodology"[TIAB] OR "narrative"[TIAB] OR "scoping"[TIAB] OR "systematic"[TIAB]) AND ("search"[TIAB] OR "searched"[TIAB] OR "searches"[TIAB] OR "studies"[TIAB]) AND ("cinahl"[TIAB] OR "cochrane"[TIAB] OR "embase"[TIAB] OR "psycinfo"[TIAB] OR "pubmed"[TIAB] OR "medline"[TIAB] OR "scopus"[TIAB] OR "web

	science"[TIAB] OR "bibliographic review"[TIAB:~1] OR "bibliographic reviews"[TIAB:~1] OR "literature review"[TIAB:~1] OR "literature reviews"[TIAB:~1] OR "literature search"[TIAB:~1] OR "literature searches"[TIAB:~1] OR "narrative review"[TIAB:~1] OR "narrative reviews"[TIAB:~1] OR "qualitative review"[TIAB:~1] OR "qualitative reviews"[TIAB:~1] OR "quantitative review"[TIAB] OR "quantitative reviews"[TIAB])) OR "comprehensive review"[TIAB] OR "comprehensive reviews"[TIAB] OR "comprehensive search"[TIAB] OR "comprehensive searches"[TIAB] OR "critical review"[TIAB] OR "critical reviews" [TIAB] OR (("electronic database"[TIAB:~1] OR "electronic databases"[TIAB:~1] OR "databases searched"[TIAB:~3]) AND (eligibility[tiab] OR excluded[tiab] OR exclusion[tiab] OR included[tiab] OR inclusion[tiab])) OR "evidence assessment"[TIAB] OR "evidence review"[TIAB] OR "exploratory review"[TIAB] OR "framework synthesis"[TIAB] OR "Integrated review"[TIAB] OR "integrated reviews"[TIAB] OR "integrative review"[TIAB:~1] OR "integrative reviews"[TIAB:~1] OR "mapping review"[TIAB:~1] OR "meta-review"[TIAB:~1] OR "meta-synthesis"[TIAB:~1] OR "methodology review"[TIAB:~1] OR ("mixed methods"[TIAB:~0] AND "methods review"[TIAB:~1]) OR ("mixed methods"[TIAB:~0] AND "methods synthesis"[TIAB:~1]) OR "overview reviews"[TIAB:~4] OR ("PRISMA"[TIAB] AND "preferred"[TIAB]) OR "PRISMA-P"[TIAB:~0] OR "prognostic review"[TIAB:~1] OR "psychometric review"[TIAB:~1] OR ("rapid evidence"[TIAB:~0] AND "evidence assessment"[TIAB:~0]) OR "rapid realist"[TIAB:~0] OR "rapid review"[TIAB:~1] OR "rapid reviews"[TIAB:~1] OR "realist review"[TIAB:~1] OR "review of reviews"[TIAB:~1] OR "scoping review"[TIAB:~1] OR "scoping reviews"[TIAB:~1] OR "scoping study"[TIAB:~1] OR ("state art "[TIAB:~2] AND "art review"[TIAB:~1]) OR "systematic evidence map"[TIAB] OR "systematic mapping"[TIAB:~1] OR "systematic literature"[TIAB:~1] OR "systematic Medline"[TIAB:~2] OR "systematic PubMed"[TIAB:~2] OR "systematic review"[TIAB:~1] OR "systematic reviews"[TIAB:~1] OR "systematic search"[TIAB:~1] OR "systematic searches"[TIAB:~1] OR "systematical review"[TIAB:~1] OR "systematical reviews"[TIAB:~1] OR "systematically identified"[TIAB:~1] OR "systematically review"[TIAB:~1] OR "systematically reviewed"[TIAB:~1] OR "umbrella review"[TIAB:~1] OR "umbrella reviews"[TIAB:~1] OR "Cochrane Database Syst Rev"[ta] OR "evid rep technol assess full rep"[Journal] OR "evid rep technol assess summ"[Journal])
Other filters	• Last 10 years: 2013 to Present<br>• Age: infant birth-6 months

Table S2. Excluded reviews

Reference [APA]	Reason for exclusion
1. Papageorghiou AT, Kennedy SH, Salomon LJ, Altman DG, Ohuma EO, Stones W, Villar J (2018). The intergrowth-21st fetal growth standards: toward the global integration of pregnancy and pediatric care.. American Journal of Obstetrics & Gynecology, 218(2S), S630-S640. https://dx.doi.org/10.1016/j.ajog.2018.01.011	No mention of maternal nutritional status and associations
2. Dewidar, O., John, J., Baqar, A., Madani, M. T., Saad, A., Riddle, A., Ota, E., Kung'u, J. K., Arabi, M., Raut, M. K., Klobodu, S. S., Rowe, S., Hatchard, J., Busch-Hallen, J., Jalal, C., Wuehler, S., & Welch, V. (2023). Effectiveness of nutrition counseling for pregnant women in low- and middle-income countries to improve maternal and infant behavioral, nutritional, and health outcomes: A systematic review. Campbell systematic reviews, 19(4), e1361. https://doi.org/10.1002/cl2.1361	No mention of maternal nutritional status and associations
3. Nguyen G, Boath A & Heslehurst N (2023). Addressing inequalities and improving maternal and infant outcomes: the potential power of nutritional interventions across the reproductive cycle.. Proceedings of the Nutrition Society, 82(3), 241-252. https://dx.doi.org/10.1017/S002966512300006X	HIC evidence (United Kingdom)
4. Venkataramani M, Ogunwole SM, Caulfield LE, Sharma R, Zhang A, Gross SM, Bennett WL (2022). Maternal, infant, and child health outcomes associated with the special supplemental nutrition program for women, infants, and children : a systematic review.. Annals of Internal Medicine, 175(10), 1411-1422. https://dx.doi.org/10.7326/M22-0604	HIC evidence (United States of America)
5. Yu Z, Han S, Zhu J, Sun X, Ji C & Guo X (2013). Pre-pregnancy body mass index in relation to infant birth weight and offspring overweight/obesity: a systematic review and meta-analysis.. PLoS ONE [Electronic Resource], 8(4), e61627. https://dx.doi.org/10.1371/journal.pone.0061627	HIC evidence - Only one study from LMICs (Pakistan) reported an association of low maternal GWG with LBW (n=144)
6. Daiy K, Harries V, Nyhan K & Marcinkowska UM (2022). Maternal weight status and the composition of the human milk microbiome: a scoping review.. PLoS ONE [Electronic Resource], 17(10), e0274950. https://dx.doi.org/10.1371/journal.pone.0274950	HIC evidence - Only one study from LMICs (Guatemala), not reporting on fetal, neonatal or infant outcomes
7. Hu, Z., Tang, L., & Xu, H. L. (2018). Maternal Vitamin D Deficiency and the Risk of Small for Gestational Age: A Meta-analysis. Iranian journal of public health, 47(12), 1785–1795.	HIC evidence - Only 1 one study form LMICs (Brazil) reported
8. Grieger, J. A., & Clifton, V. L. (2014). A review of the impact of dietary intakes in human pregnancy on infant birthweight. Nutrients, 7(1), 153–178. https://doi.org/10.3390/nu7010153	HIC evidence
9. Wu D, Li Y, Chen L, Klein M, Franke B, Chen J & Buitelaar J (2023). Maternal gestational weight gain and offspring's neurodevelopmental outcomes: a systematic review and meta-analysis..	HIC evidence only

Neuroscience & Biobehavioral Reviews, 153, 105360. https://dx.doi.org/10.1016/j.neubiorev.2023.105360	
10. Chen, Y., Zhu, B., Wu, X., Li, S., & Tao, F. (2017). Association between maternal vitamin D deficiency and small for gestational age: evidence from a meta-analysis of prospective cohort studies. BMJ open, 7(8), e016404. https://doi.org/10.1136/bmjopen-2017-016404	HIC evidence only
11. Zhang, Q., Wang, Y., Xin, X., Zhang, Y., Liu, D., Peng, Z., He, Y., Xu, J., & Ma, X. (2017). Effect of folic acid supplementation on preterm delivery and small for gestational age births: A systematic review and meta-analysis. Reproductive toxicology (Elmsford, N.Y.), 67, 35–41. https://doi.org/10.1016/j.reprotox.2016.11.012	HIC evidence only
12. Chen, L. W., Wu, Y., Neelakantan, N., Chong, M. F., Pan, A., & van Dam, R. M. (2016). Maternal caffeine intake during pregnancy and risk of pregnancy loss: a categorical and dose-response meta-analysis of prospective studies. *Public health nutrition*, *19*(7), 1233–1244. https://doi.org/10.1017/S1368980015002463	HIC evidence (USA, UK, Europe)
13. Caulfield, L. E., Bennett, W. L., Gross, S. M., Hurley, K. M., Ogunwole, S. M., Venkataramani, M., Lerman, J. L., Zhang, A., Sharma, R., & Bass, E. B. (2022). Maternal and Child Outcomes Associated With the Special Supplemental Nutrition Program for Women, Infants, and Children (WIC). Agency for Healthcare Research and Quality (US).	HIC evidence (USA)
14. Raab R, Michel S, Gunther J, Hoffmann J, Stecher L & Hauner H (2021). Associations between lifestyle interventions during pregnancy and childhood weight and growth: a systematic review and meta-analysis.. International Journal of Behavioral Nutrition & Physical Activity, 18(1), 8. https://dx.doi.org/10.1186/s12966-020-01075-7	HIC evidence
15. Goldstein RF, Abell SK, Ranasinha S, Misso M, Boyle JA, Black MH, Teede HJ (2017). Association of gestational weight gain with maternal and infant outcomes: a systematic review and meta-analysis.. JAMA, 317(21), 2207-2225. https://dx.doi.org/10.1001/jama.2017.3635	HIC evidence
16. Goldstein RF, Abell SK, Ranasinha S, Misso ML, Boyle JA, Harrison CL, Teede HJ (2018). Gestational weight gain across continents and ethnicity: systematic review and meta-analysis of maternal and infant outcomes in more than one million women.. BMC Medicine, 16(1), 153. https://dx.doi.org/10.1186/s12916-018-1128-1	HIC evidence
17. Raghavan, R., Dreibelbis, C., Kingshipp, B. L., Wong, Y. P., Abrams, B., Gernand, A. D., Rasmussen, K. M., Siega-Riz, A. M., Stang, J., Casavale, K. O., Spahn, J. M., & Stoody, E. E. (2019). Dietary patterns before and during pregnancy and maternal outcomes: a systematic review. The American journal of clinical nutrition, 109(Suppl_7), 705S–728S. https://doi.org/10.1093/ajcn/nqy216	HIC evidence
18. Nagpal, T. S., Souza, S. C. S., Moffat, M., Hayes, L., Nuyts, T., Liu, R. H., Bogaerts, A., Dervis, S., Piccinini-Vallis, H., Adamo, K. B., & Heslehurst, N. (2022). Does prepregnancy weight change have an effect on subsequent pregnancy health outcomes? A systematic review and meta-analysis. Obesity	HIC evidence

reviews : an official journal of the International Association for the Study of Obesity, 23(1), e13324. https://doi.org/10.1111/obr.13324	
19. Xu, J., Wang, H., Bian, J., Xu, M., Jiang, N., Luo, W., Zu, P., Yin, W., & Zhu, P. (2024). Association between the Maternal Mediterranean Diet and Perinatal Outcomes: A Systematic Review and Meta-Analysis. *Advances in nutrition (Bethesda, Md.)*, *15*(2), 100159. https://doi.org/10.1016/j.advnut.2023.100159	HIC evidence
20. Raghavan, R., Dreibelbis, C., Kingshipp, B. L., Wong, Y. P., Abrams, B., Gernand, A. D., ... & Stoody, E. E. (2019). Dietary patterns before and during pregnancy and maternal outcomes: a systematic review. *The American journal of clinical nutrition*, *109*, 705S-728S.	HIC evidence
21. Mohamed HJJ, Loy SL, Mitra AK, Kaur S, Teoh AN, Rahman SHA & Amarra MS (2022). Maternal diet, nutritional status and infant birth weight in malaysia: a scoping review.. BMC Pregnancy & Childbirth, 22(1), 294. https://dx.doi.org/10.1186/s12884-022-04616-z	Only UMIC evidence (Malaysia)
22. Slack, E., Rankin, J., Jones, D., & Heslehurst, N. (2018). Effects of maternal anthropometrics on pregnancy outcomes in South Asian women: a systematic review. Obesity reviews : an official journal of the International Association for the Study of Obesity, 19(4), 485–500. https://doi.org/10.1111/obr.12636	Only UMIC evidence (South Africa)<br>Narrative review; no tallies
23. Dorsamy, V., Bagwandeen, C., & Moodley, J. (2022). The prevalence, risk factors and outcomes of anaemia in South African pregnant women: a systematic review and meta-analysis. Systematic reviews, 11(1), 16. https://doi.org/10.1186/s13643-022-01884-w	Only UMIC evidence (South African cohort)
24. Marshall NE, Abrams B, Barbour LA, Catalano P, Christian P, Friedman JE, Thornburg KL (2022). The importance of nutrition in pregnancy and lactation: lifelong consequences.. American Journal of Obstetrics & Gynecology, 226(5), 607-632. https://dx.doi.org/10.1016/j.ajog.2021.12.035	Review does not report settings where studies where conducted
25. Ferreira LB, Lobo CV, Miranda AEDS, Carvalho BDC & Santos LCD (2022). Dietary patterns during pregnancy and gestational weight gain: a systematic review.. Revista Brasileira de Ginecologia e Obstetricia, 44(5), 540-547. https://dx.doi.org/10.1055/s-0042-1744290	No fetal, neonatal or infant outcomes
26. Arzhang P, Ramezan M, Borazjani M, Jamshidi S, Bavani NG, Rahmanabadi A & Bagheri A (2022). The association between food insecurity and gestational weight gain: a systematic review and meta-analysis.. Appetite, 176, 106124. https://dx.doi.org/10.1016/j.appet.2022.106124	No fetal, neonatal or infant outcomes
27. Opsahl JO, Moen GH, Qvigstad E, Bottcher Y, Birkeland KI & Sommer C (2021). Epigenetic signatures associated with maternal body mass index or gestational weight gain: a systematic review.. Journal of Developmental Origins of Health and Disease, 12(3), 373-383. https://dx.doi.org/10.1017/S2040174420000811	No fetal, neonatal or infant outcomes
28. Abe, S. K., Balogun, O. O., Ota, E., Takahashi, K., & Mori, R. (2016). Supplementation with multiple micronutrients for breastfeeding women for improving outcomes for the mother and baby. *The Cochrane database of systematic reviews*, *2*(2), CD010647. https://doi.org/10.1002/14651858.CD010647.pub2	No fetal, neonatal or infant outcomes

29. Szostak-Wegierek, D. (2014). Intrauterine nutrition: long-term consequences for vascular health. *International Journal of Women's Health*, *6*, 647–656. https://doi.org/10.2147/IJWH.S48751	No fetal, neonatal or infant outcomes<br>Narrative review; no tallies
30. Teede HJ, Bailey C, Moran LJ, Bahri Khomami M, Enticott J, Ranasinha S, Harrison CL (2022). Association of antenatal diet and physical activity-based interventions with gestational weight gain and pregnancy outcomes: a systematic review and meta-analysis.. JAMA Internal Medicine, 182(2), 106-114. https://dx.doi.org/10.1001/jamainternmed.2021.6373	Maternal obesity and related themes (e.g., Bariatric surgery)
31. Pari-Keener M, Gallo S, Stahnke B, McDermid JM, Al-Nimr RI, Moreschi JM, Cheng FW (2020). Maternal and infant health outcomes associated with medical nutrition therapy by registered dietitian nutritionists in pregnant women with malnutrition: an evidence analysis center systematic review.. Journal of the Academy of Nutrition & Dietetics, 120(10), 1730-1744. https://dx.doi.org/10.1016/j.jand.2019.10.024	Maternal obesity and related themes (e.g., Bariatric surgery)
32. Vezina-Im LA, Nicklas TA & Baranowski T (2018). Intergenerational effects of health issues among women of childbearing age: a review of the recent literature.. Current Nutrition Reports, 7(4), 274-285. https://dx.doi.org/10.1007/s13668-018-0246-x	Maternal obesity and related themes (e.g., Bariatric surgery)
33. Szostak-Wegierek D. Intrauterine nutrition: long-term consequences for vascular health. Int J Womens Health. 2014 Jul 11;6:647-56. doi: 10.2147/IJWH.S48751. PMID: 25050077; PMCID: PMC4103922.	Maternal obesity and related themes,<br>No fetal, neonatal or infant outcomes
34. Dalfra', M. G., Burlina, S., & Lapolla, A. (2022). Weight gain during pregnancy: A narrative review on the recent evidences. Diabetes research and clinical practice, 188, 109913. https://doi.org/10.1016/j.diabres.2022.109913	Maternal obesity and related themes
35. Liu, P., Xu, L., Wang, Y., Zhang, Y., Du, Y., Sun, Y., & Wang, Z. (2016). Association between perinatal outcomes and maternal pre-pregnancy body mass index. Obesity reviews : an official journal of the International Association for the Study of Obesity, 17(11), 1091–1102. https://doi.org/10.1111/obr.12455	Maternal obesity and related themes
36. Grissom NM & Reyes TM (2013). Gestational overgrowth and undergrowth affect neurodevelopment: similarities and differences from behavior to epigenetics.. International Journal of Developmental Neuroscience, 31(6), 406-14. https://dx.doi.org/10.1016/j.ijdevneu.2012.11.006	Review focused on mechanistic pathways in animal and human models
37. Diniz, M. S., Magalhães, C. C., Tocantins, C., Grilo, L. F., Teixeira, J., & Pereira, S. P. (2023). Nurturing through Nutrition: Exploring the Role of Antioxidants in Maternal Diet during Pregnancy to Mitigate Developmental Programming of Chronic Diseases. Nutrients, 15(21), 4623. https://doi.org/10.3390/nu15214623	Review focused on mechanistic pathways in animal and human models
38. Domínguez-Perles, R., Gil-Izquierdo, A., Ferreres, F., & Medina, S. (2019). Update on oxidative stress and inflammation in pregnant women, unborn children (nasciturus), and newborns–Nutritional and dietary effects. Free Radical Biology and Medicine, 142, 38-51.	Narrative review, HIC evidence

39. Warland, J., Mitchell, E. A., & O'Brien, L. M. (2017). Novel strategies to prevent stillbirth. Seminars in fetal & neonatal medicine, 22(3), 146–152. https://doi.org/10.1016/j.siny.2017.01.005	Narrative review, HIC evidence
40. White, M. R., & Yates, D. T. (2023). Dousing the flame: reviewing the mechanisms of inflammatory programming during stress-induced intrauterine growth restriction and the potential for ω-3 polyunsaturated fatty acid intervention. Frontiers in Physiology, 14, 1250134.	Narrative review, HIC evidence
41. Papathakis PC, Singh LN & Manary MJ (2016). How maternal malnutrition affects linear growth and development in the offspring.. Molecular & Cellular Endocrinology, 435, 40-47. https://dx.doi.org/10.1016/j.mce.2016.01.024	Narrative review, included studies duplicated with other included reviews
42. Santos-Antonio G, Alvis-Chirinos K, Aguilar-Esenarro L, Bautista-Olortegui W, Velarde-Delgado P & Aramburu A (2020). Gestational weight gain as a predictor of macrosomia and low birth weight: a systematic review.. Revista Peruana de Medicina Experimental y Salud Publica, 37(3), 403-411. https://dx.doi.org/10.17843/rpmesp.2020.373.4919	Narrative review; no tallies
43. Gilani, S., & Janssen, P. (2020). Maternal Vitamin D Levels During Pregnancy and Their Effects on Maternal-Fetal Outcomes: A Systematic Review. *Journal of obstetrics and gynaecology Canada : JOGC = Journal d'obstetrique et gynecologie du Canada : JOGC*, *42*(9), 1129–1137. https://doi.org/10.1016/j.jogc.2019.09.013	Narrative review; no tallies
44. Zerfu, T. A., & Ayele, H. T. (2013). Micronutrients and pregnancy; effect of supplementation on pregnancy and pregnancy outcomes: a systematic review. *Nutrition journal*, *12*, 20. https://doi.org/10.1186/1475-2891-12-20	Narrative review; no tallies
45. Weckman, A. M., Ngai, M., Wright, J., McDonald, C. R., & Kain, K. C. (2019). The impact of infection in pregnancy on placental vascular development and adverse birth outcomes. *Frontiers in microbiology*, *10*, 1924.	Narrative review focused on mechanistic pathways in animal and human models
46. Uriza, B., JA, A. M., Cavagnari, B. M., González, C., Tellez, C., Brambila, C., ... & Molina, L. (2020). Non-caloric sweeteners in women of reproductive age-A consensus document. *Nutricion Hospitalaria*, *37*(1), 211-222.	Not a review – Consensus document
47. Nadhiroh, S. R., Micheala, F., Tung, S. E. H., & Kustiawan, T. C. (2023). Association between maternal anemia and stunting in infants and children aged 0-60 months: A systematic literature review. Nutrition (Burbank, Los Angeles County, Calif.), 115, 112094. https://doi.org/10.1016/j.nut.2023.112094	Outcomes reported in review differ from individual studies
48. Firouzabadi, F. D., Shab-Bidar, S., & Jayedi, A. (2022). The effects of omega-3 polyunsaturated fatty acids supplementation in pregnancy, lactation, and infancy: An umbrella review of meta-analyses of randomized trials. *Pharmacological research*, *177*, 106100. https://doi.org/10.1016/j.phrs.2022.106100	Primary studies duplicated with included Cochrane review or other larger reviews
49. Agarwal, S., Kovilam, O., & Agrawal, D. K. (2018). Vitamin D and its impact on maternal-fetal outcomes in pregnancy: A critical review. *Critical reviews in food science and nutrition*, *58*(5), 755–769. https://doi.org/10.1080/10408398.2016.1220915	Primary studies duplicated with included Cochrane review or other larger reviews

50. Dewidar, O., John, J., Baqar, A., Madani, M. T., Saad, A., Riddle, A., Ota, E., Kung'u, J. K., Arabi, M., Raut, M. K., Klobodu, S. S., Rowe, S., Hatchard, J., Busch-Hallen, J., Jalal, C., Wuehler, S., & Welch, V. (2023). Effectiveness of nutrition counseling for pregnant women in low- and middle-income countries to improve maternal and infant behavioral, nutritional, and health outcomes: A systematic review. *Campbell systematic reviews*, *19*(4), e1361. https://doi.org/10.1002/cl2.1361	Primary studies duplicated with included Cochrane review or other larger reviews

Table S3. Characteristics of included reviews

Pre-conception or Pre-pregnancy
Early Pregnancy
Late Pregnancy
Post-partum or Lactation
Time during pregnancy not specified

Review Author, year	Study type	Maternal nutritional indicators or interventions	Fetal, neonatal or infant outcomes significantly associated
**Maternal diet**			
Murphy 2014 [1]	SR	Energy-adjusted fruit and vegetable intake during pregnancy	BW, SGA
Abdollahi et al, 2021 [2]	SR	Maternal adherence to healthy diet	BW length, head circumference, stillbirth, SGA, LBW, PTB
Seid 2023 [3]	SR-MA	Inadequate dietary diversity	LBW
**Anthropometry**			
Dean 2014 [4]	SR-MA	Pre-pregnancy underweight	PTB, SGA, (Non-significant effect on LBW)
Rahman 2015 [5]	SR-MA	Pre-pregnancy underweight	PTB, LBW, SGA (Non-significant effect on stillbirths)
Goto 2016 [6]	SR-MA	Maternal height, weight, BMI, weight gain	SGA
Wrottesley 2016 [7]	Review	Maternal MUAC<23cm, Maternal height<156cm<br>100 mg of iron/day<br>MMN supplementation<br>Protein-energy supplementation	LBW<br>Birthweight<br>BW<br>Head circumference

Accrombessi 2017 [8]	Scoping review	Maternal undernutrition (low BMI during pregnancy and low GWG)	IUGR
Akombi 2017 [9]	Narrative review	Maternal underweight, low maternal height, maternal diet	Stunting, wasting and underweight in children
Perumal et al, 2023 [10]	SR-MA	Severely/moderately inadequate GWG	Stillbirth, PTB, LBW, Neonatal death
Yang 2023 [11]	SR	Maternal undernutrition (low pre-pregnancy BMI, low GWG, anemia)	IUGR
**Anemia/Haemoglobin (Hb) levels**			
Rahman 2016 [12]	SR-MA	Maternal anemia	LBW, perinatal and neonatal mortality
Dewey 2017 [13]	Narrative review	Hb concentration/anemia in the 1st and 2nd trimesters	Stillbirth, PTB, SGA, LBW
		Hb concentration/anemia in the 3rd trimester	
Rahmati 2017 [14]	SR-MA	Maternal anemia	Infant LBW in the 1st, 2nd and 3rd trimester of pregnancy
Figuerido 2018 [15]	SR-MA	Maternal anemia	LBW
Rahman 2020 [16]	SR-MA	Maternal anemia	PTB, LBW, perinatal mortality, neonatal mortality
**Micronutrient status**			
Sole-Navais 2016 [17]	Review	Low folate and vitamin B12 intake	SGA
Sukumar 2016 [18]	SR-MA	Low maternal or cord blood B12 levels	LBW/SGA
Rogne 2017 [19]	SR-MA (Individual patient data)	Maternal vitamin B12 deficiency	LBW, PTB
van der Pligt 2018 [20]	SR	Vitamin D deficiency	LBW, SGA
Dos Santos 2023[21]	SR	Vitamin D levels	PTB, BW
Reyes 2024 [22]	Narrative review	Breastmilk micronutrients	Infant growth outcomes (WAZ, HAZ, WHZ, BMI-for-age, growth velocity)
**Micronutrient supplementation**			
Bhutta & Das 2014 [23]	Chapter	Daily iron supplementation<br>MMN supplementation<br>BEP supplementation	LBW<br>LBW, SGA<br>LBW, SGA, neonatal mortality
Gresham 2014 [24]	SR-MA	Food/fortified food supplementation + counselling	BW, LBW
Buppasiri 2015 [25]	Cochrane SR	Calcium supplementation	PTB, LBW
Cantor 2015 [26]	SR	Iron supplementation <20 wks	SGA, LBW, PTB, Apgar scores, perinatal mortality

McCauley 2015 [27]	Cochrane SR	Vitamin A supplementation (during pregnancy – trimester not specified)	Stillbirth, PTB, neonatal mortality, perinatal mortality
Ota 2015 [28]	Cochrane SR	Antenatal dietary education	BW in under-nourished women, LBW, PTB (No effect on mean gestational age)
		BEP supplementation	Stillbirth, SGA, BW (No effect on neonatal mortality, head circumference, PTB, outcomes in infancy)
Peña-Rosas 2015 [29]	Cochrane SR	Iron supplementation	LBW, BW, PTB, congenital anomalies, neonatal mortality
Rumbold 2015a [30]	Cochrane SR	Vitamin E supplementation	IUGR, stillbirth, PROM, PTB, neonatal death
Rumbold 2015b [31]	Cochrane SR	Vitamin C supplementation combined with other micronutrients<br>Vitamin C supplementation	IUGR, stillbirth, PROM<br>BW, PTB, neonatal or perinatal death
Saccone 2015 [32]	SR-MA	Omega-3 fatty acid supplementation	Neonatal outcomes
Stevens 2015 [33]	SR-MA	BEP supplementation	BW, LBW, SGA, neonatal mortality, length, head circumference
Balogun 2016 [34]	Cochrane SR	Multivitamin + iron/folate<br>micronutrient supplementation (vitamin C, vitamin A, antioxidant vitamins)	Stillbirth (No effect on total fetal loss, early or late miscarriage)
Oliveira 2016 [35]	Cochrane SR	Vitamin A supplementation (during lactation)	Infant mortality, neonatal infections
Saccone 2016 [36]	Review	Omega-3 fatty acid supplementation	IUGR, perinatal death
Amegah 2017 [37]	SR-MA	Vitamin D insufficieny/supplementation	PTB, Apgar scores (No effects on incidence of short gestational length, spontaneous abortion, stillbirth)
Haider & Bhutta 2017 [38]	Cochrane SR	MMN supplementation	LBW, SGA, PTB
Das 2018 [39]	Cochrane SR	Lipid-based supplements	BW, length at birth, LBW, SGA, newborn stunting
Hofmeyr 2018 [40]	Cochrane SR	Calcium supplementation (high dose >/=1g, low-dose <1g)	Miscarriage, stillbirth, neonatal death, PTB, LBW, SGA
Abraha 2019 [41]	Overview of reviews	Iron therapies	PTB, LBW, infant mortality
Bourassa 2019 [42] [Smith et al. 2017, Keats et al. 2019]	Umbrella review	Multivitamin + iron/folate	Stillbirth, LBW, SGA
		MMN supplementation	

Palacios 2019 [43]	Cochrane SR	Vitamin D and calcium supplementation	PTB
Adu -Afarwhuah et al, 2020 [44]	Narrative review	Prenatal SQ-LNS	Fetal growth, LBW
Lassi 2020 [45, 46]	SR	Food distribution programs<br>BEP supplementation	PTB, SGA, LBW, perinatal mortality, wasting, stillbirth, BW, LBW, SGA, neonatal mortality
Daly 2022 [47]<br>[De-Regil et al. 2015]	Umbrella review	Folate and other vitamin supplementation	Neural tube defects, miscarriage, anomaly-related terminated, LBW, congenital heart defects or other birth defects
Carducci 2021c[48]	Cochrane SR	Zinc supplementation	Stillbirth, PTB, LBW, SGA or perinatal death
Zhao 2022[49]	SR and dose-response MA	Vitamin D supplementation <20 wks<br>Vitamin D supplementation >20 wks	IUGR, LBW, PTB, SGA
Ciulei 2023 [50]	Narrative review	BEP supplementation<br>Lipid-based supplements	Stillbirth<br>BW, LBW, SGA, neonatal mortality<br>BW, length at birth
Hansen 2023 [51]	SR-MA	Daily oral iron supplementation	SGA, LBW, PTB

Legend: BEP: balanced energy protein, BMI: body-mass index, BW: birthweight, GWG: gestational weight gain, HAZ: height-for-age z-scores, Hb: hemoglobin, IUGR: intra-uterine growth restriction, LBW: low birth weight, MA: meta-analysis, mg: milligram, MMN: multiple micronutrients, MNP: multiple micronutrient powder, MUAC: mid-upper arm circumference, PTB: preterm birth, SGA: small-for-gestational age, SR: systematic review, SQ-LNS: small-quantity lipid-based nutrient supplements, WAZ: weight-for-age z-scores, WHZ: weight-for-height z-scores

Table S4. PRISMA Checklist

Section and Topic	Item #	Checklist item	Location where item is reported
**TITLE**			
Title	1	Identify the report as a systematic review.	Title – Overview of reviews
**ABSTRACT**			
Abstract	2	See the PRISMA 2020 for Abstracts checklist.	9-28
**INTRODUCTION**			
Rationale	3	Describe the rationale for the review in the context of existing knowledge.	71-81
Objectives	4	Provide an explicit statement of the objective(s) or question(s) the review addresses.	82-92
**METHODS**			
Eligibility criteria	5	Specify the inclusion and exclusion criteria for the review and how studies were grouped for the syntheses.	Table 1, 112-118
Information sources	6	Specify all databases, registers, websites, organisations, reference lists and other sources searched or consulted to identify studies. Specify the date when each source was last searched or consulted.	100-103
Search strategy	7	Present the full search strategies for all databases, registers and websites, including any filters and limits used.	Table S1
Selection process	8	Specify the methods used to decide whether a study met the inclusion criteria of the review, including how many reviewers screened each record and each report retrieved, whether they worked independently, and if applicable, details of automation tools used in the process.	119-121
Data collection process	9	Specify the methods used to collect data from reports, including how many reviewers collected data from each report, whether they worked independently, any processes for obtaining or confirming data from study investigators, and if applicable, details of automation tools used in the process.	129-131
Data items	10a	List and define all outcomes for which data were sought. Specify whether all results that were compatible with each outcome domain in each study were sought (e.g. for all measures, time points, analyses), and if not, the methods used to decide which results to collect.	Table 1
	10b	List and define all other variables for which data were sought (e.g. participant and intervention characteristics, funding sources). Describe any assumptions made about any missing or unclear information.	123-143
Study risk of bias assessment	11	Specify the methods used to assess risk of bias in the included studies, including details of the tool(s) used, how many reviewers assessed each study and whether they worked independently, and if applicable, details of automation tools used in the process.	137-143
Effect measures	12	Specify for each outcome the effect measure(s) (e.g. risk ratio, mean difference) used in the synthesis or presentation of results.	123-126
Synthesis methods	13a	Describe the processes used to decide which studies were eligible for each synthesis (e.g. tabulating the study intervention characteristics and comparing against the planned groups for each synthesis (item #5)).	Table S.3.
	13b	Describe any methods required to prepare the data for presentation or synthesis, such as handling of missing summary statistics, or data conversions.	126-137
	13c	Describe any methods used to tabulate or visually display results of individual studies and syntheses.	134-137
	13d	Describe any methods used to synthesize results and provide a rationale for the choice(s). If meta-analysis was performed, describe the model(s), method(s) to identify the presence and extent of statistical heterogeneity, and software package(s) used.	123-129

	13e	Describe any methods used to explore possible causes of heterogeneity among study results (e.g. subgroup analysis, meta-regression).	123-126
	13f	Describe any sensitivity analyses conducted to assess robustness of the synthesized results.	138-143
Reporting bias assessment	14	Describe any methods used to assess risk of bias due to missing results in a synthesis (arising from reporting biases).	Table 2
Certainty assessment	15	Describe any methods used to assess certainty (or confidence) in the body of evidence for an outcome.	138-143
**RESULTS**			
Study selection	16a	Describe the results of the search and selection process, from the number of records identified in the search to the number of studies included in the review, ideally using a flow diagram.	Figure 2
	16b	Cite studies that might appear to meet the inclusion criteria, but which were excluded, and explain why they were excluded.	Table S.2.
Study characteristics	17	Cite each included study and present its characteristics.	Table S.3.
Risk of bias in studies	18	Present assessments of risk of bias for each included study.	Table 2
Results of individual studies	19	For all outcomes, present, for each study: (a) summary statistics for each group (where appropriate) and (b) an effect estimate and its precision (e.g. confidence/credible interval), ideally using structured tables or plots.	Table 2
Results of syntheses	20a	For each synthesis, briefly summarise the characteristics and risk of bias among contributing studies.	Table 2
	20b	Present results of all statistical syntheses conducted. If meta-analysis was done, present for each the summary estimate and its precision (e.g. confidence/credible interval) and measures of statistical heterogeneity. If comparing groups, describe the direction of the effect.	Table 2
	20c	Present results of all investigations of possible causes of heterogeneity among study results.	N/A
	20d	Present results of all sensitivity analyses conducted to assess the robustness of the synthesized results.	N/A
Reporting biases	21	Present assessments of risk of bias due to missing results (arising from reporting biases) for each synthesis assessed.	Table 2
Certainty of evidence	22	Present assessments of certainty (or confidence) in the body of evidence for each outcome assessed.	Table 2
**DISCUSSION**			
Discussion	23a	Provide a general interpretation of the results in the context of other evidence.	386-390
	23b	Discuss any limitations of the evidence included in the review.	356-359, 391-399, 411-414, 492-495 496-503,
	23c	Discuss any limitations of the review processes used.	496-503
	23d	Discuss implications of the results for practice, policy, and future research.	427-428, 442-449 514-519,
**OTHER INFORMATION**			
Registration and protocol	24a	Provide registration information for the review, including register name and registration number, or state that the review was not registered.	98-100
	24b	Indicate where the review protocol can be accessed, or state that a protocol was not prepared.	98-100

	24c	Describe and explain any amendments to information provided at registration or in the protocol.	104-105
Support	25	Describe sources of financial or non-financial support for the review, and the role of the funders or sponsors in the review.	529-530
Competing interests	26	Declare any competing interests of review authors.	537-539
Availability of data, code and other materials	27	Report which of the following are publicly available and where they can be found: template data collection forms; data extracted from included studies; data used for all analyses; analytic code; any other materials used in the review.	534-535

From: Page MJ, McKenzie JE, Bossuyt PM, Boutron I, Hoffmann TC, Mulrow CD, et al. The PRISMA 2020 statement: an updated guideline for reporting systematic reviews. BMJ 2021;372:n71. doi: 10.1136/bmj.n71

References

1Murphy MM, Stettler N, Smith KM, Reiss R. Associations of consumption of fruits and vegetables during pregnancy with infant birth weight or small for gestational age births: a systematic review of the literature. Int J Womens Health. 2014;6:899-912.

2Abdollahi S, Soltani S, de Souza RJ, Forbes SC, Toupchian O, Salehi-Abargouei A. Associations between Maternal Dietary Patterns and Perinatal Outcomes: A Systematic Review and Meta-Analysis of Cohort Studies. Adv Nutr. 2021 Jul 30;12(4):1332-52.

3Seid A, Dugassa Fufa D, Weldeyohannes M, Tadesse Z, Fenta SL, Bitew ZW, Dessie G. Inadequate dietary diversity during pregnancy increases the risk of maternal anemia and low birth weight in Africa: A systematic review and meta-analysis. Food Sci Nutr. 2023 Jul;11(7):3706-17.

4Dean SV, Lassi ZS, Imam AM, Bhutta ZA. Preconception care: nutritional risks and interventions. Reprod Health. 2014 Sep 26;11 Suppl 3(Suppl 3):S3.

5Rahman MM, Abe SK, Kanda M, Narita S, Rahman MS, Bilano V, et al. Maternal body mass index and risk of birth and maternal health outcomes in low- and middle-income countries: a systematic review and meta-analysis. Obes Rev. 2015 Sep;16(9):758-70.

6Goto E. Maternal anthropometry to predict small for gestational age: a meta-analysis. European Journal of Obstetrics & Gynecology and Reproductive Biology. 2016;203:193-98.

7Wrottesley SV, Lamper C, Pisa PT. Review of the importance of nutrition during the first 1000 days: maternal nutritional status and its associations with fetal growth and birth, neonatal and infant outcomes among African women. J Dev Orig Health Dis. 2016 Apr;7(2):144-62.

8Accrombessi M, Zeitlin J, Massougbodji A, Cot M, Briand V. What Do We Know about Risk Factors for Fetal Growth Restriction in Africa at the Time of Sustainable Development Goals? A Scoping Review. Paediatr Perinat Epidemiol. 2018 Mar;32(2):184-96.

9Akombi BJ, Agho KE, Hall JJ, Wali N, Renzaho AMN, Merom D. Stunting, Wasting and Underweight in Sub-Saharan Africa: A Systematic Review. Int J Environ Res Public Health. 2017 Aug 1;14(8).

10Perumal N, Wang D, Darling AM, Liu E, Wang M, Ahmed T, et al. Suboptimal gestational weight gain and neonatal outcomes in low and middle income countries: individual participant data meta-analysis. Bmj. 2023 Sep 21;382:e072249.

11Yang L, Feng L, Huang L, Li X, Qiu W, Yang K, et al. Maternal Factors for Intrauterine Growth Retardation: Systematic Review and Meta-Analysis of Observational Studies. Reprod Sci. 2023 Jun;30(6):1737-45.
12Rahman MM, Abe SK, Rahman MS, Kanda M, Narita S, Bilano V, et al. Maternal anemia and risk of adverse birth and health outcomes in low- and middle-income countries: systematic review and meta-analysis. Am J Clin Nutr. 2016 Feb;103(2):495-504.
13Dewey KG, Oaks BM. U-shaped curve for risk associated with maternal hemoglobin, iron status, or iron supplementation. Am J Clin Nutr. 2017 Dec;106(Suppl 6):1694s-702s.
14Rahmati S, Delpishe A, Azami M, Hafezi Ahmadi MR, Sayehmiri K. Maternal Anemia during pregnancy and infant low birth weight: A systematic review and Meta-analysis. Int J Reprod Biomed. 2017 Mar;15(3):125-34.
15Figueiredo A, Gomes-Filho IS, Silva RB, Pereira PPS, Mata F, Lyrio AO, et al. Maternal Anemia and Low Birth Weight: A Systematic Review and Meta-Analysis. Nutrients. 2018 May 12;10(5).
16Rahman MA, Khan MN, Rahman MM. Maternal anaemia and risk of adverse obstetric and neonatal outcomes in South Asian countries: A systematic review and meta-analysis. Public Health Pract (Oxf). 2020 Nov;1:100021.
17Solé-Navais P, Cavallé-Busquets P, Fernandez-Ballart JD, Murphy MM. Early pregnancy B vitamin status, one carbon metabolism, pregnancy outcome and child development. Biochimie. 2016 Jul;126:91-6.
18Sukumar N, Rafnsson SB, Kandala NB, Bhopal R, Yajnik CS, Saravanan P. Prevalence of vitamin B-12 insufficiency during pregnancy and its effect on offspring birth weight: a systematic review and meta-analysis. Am J Clin Nutr. 2016 May;103(5):1232-51.
19Rogne T, Tielemans MJ, Chong MF, Yajnik CS, Krishnaveni GV, Poston L, et al. Associations of Maternal Vitamin B12 Concentration in Pregnancy With the Risks of Preterm Birth and Low Birth Weight: A Systematic Review and Meta-Analysis of Individual Participant Data. Am J Epidemiol. 2017 Feb 1;185(3):212-23.
20van der Pligt P, Willcox J, Szymlek-Gay EA, Murray E, Worsley A, Daly RM. Associations of Maternal Vitamin D Deficiency with Pregnancy and Neonatal Complications in Developing Countries: A Systematic Review. Nutrients. 2018 May 18;10(5).
21dos Santos SdF, dos Reis Costa PN, Gouvêa TG, de Almeida NFA, Cardoso FdS. Influence of hypovitaminosis D during pregnancy on glycemic and lipid profile, inflammatory indicators and anthropometry of pregnant and newborn. Clinical Nutrition ESPEN. 2023 2023/04/01/;54:81-93.
22Reyes SM, Brockway MM, McDermid JM, Chan D, Granger M, Refvik R, et al. Human Milk Micronutrients and Child Growth and Body Composition in the First 2 years: A Systematic Review. Adv Nutr. 2024 Jan;15(1):100082.
23Bhutta ZA, Das JK. Interventions to address maternal and childhood undernutrition: current evidence. Nestle Nutr Inst Workshop Ser. 2014;78:59-69.
24Gresham E, Byles JE, Bisquera A, Hure AJ. Effects of dietary interventions on neonatal and infant outcomes: a systematic review and meta-analysis. Am J Clin Nutr. 2014 Nov;100(5):1298-321.
25Buppasiri P, Lumbiganon P, Thinkhamrop J, Ngamjarus C, Laopaiboon M, Medley N. Calcium supplementation (other than for preventing or treating hypertension) for improving pregnancy and infant outcomes. Cochrane Database Syst Rev. 2015 Feb 25;2015(2):Cd007079.
26Cantor AG, Bougatsos C, Dana T, Blazina I, McDonagh M. Routine iron supplementation and screening for iron deficiency anemia in pregnancy: a systematic review for the U.S. Preventive Services Task Force. Ann Intern Med. 2015 Apr 21;162(8):566-76.
27McCauley ME, van den Broek N, Dou L, Othman M. Vitamin A supplementation during pregnancy for maternal and newborn outcomes. Cochrane Database Syst Rev. 2015 Oct 27;2015(10):Cd008666.

28Ota E, Hori H, Mori R, Tobe-Gai R, Farrar D. Antenatal dietary education and supplementation to increase energy and protein intake. Cochrane Database Syst Rev. 2015 Jun 2(6):Cd000032.
29Peña-Rosas JP, De-Regil LM, Garcia-Casal MN, Dowswell T. Daily oral iron supplementation during pregnancy. Cochrane Database Syst Rev. 2015 Jul 22;2015(7):Cd004736.
30Rumbold A, Ota E, Nagata C, Shahrook S, Crowther CA. Vitamin C supplementation in pregnancy. Cochrane Database Syst Rev. 2015 Sep 29(9):Cd004072.
31Rumbold A, Ota E, Hori H, Miyazaki C, Crowther CA. Vitamin E supplementation in pregnancy. Cochrane Database Syst Rev. 2015 Sep 7;2015(9):Cd004069.
32Saccone G, Berghella V, Maruotti GM, Sarno L, Martinelli P. Omega-3 supplementation during pregnancy to prevent recurrent intrauterine growth restriction: systematic review and meta-analysis of randomized controlled trials. Ultrasound Obstet Gynecol. 2015 Dec;46(6):659-64.
33Stevens B, Buettner P, Watt K, Clough A, Brimblecombe J, Judd J. The effect of balanced protein energy supplementation in undernourished pregnant women and child physical growth in low- and middle-income countries: a systematic review and meta-analysis. Matern Child Nutr. 2015 Oct;11(4):415-32.
34Balogun OO, da Silva Lopes K, Ota E, Takemoto Y, Rumbold A, Takegata M, Mori R. Vitamin supplementation for preventing miscarriage. Cochrane Database Syst Rev. 2016 May 6;2016(5):Cd004073.
35Oliveira JM, Allert R, East CE. Vitamin A supplementation for postpartum women. Cochrane Database Syst Rev. 2016 Mar 25;3(3):Cd005944.
36Saccone G, Saccone I, Berghella V. Omega-3 long-chain polyunsaturated fatty acids and fish oil supplementation during pregnancy: which evidence? J Matern Fetal Neonatal Med. 2016;29(15):2389-97.
37Amegah AK, Klevor MK, Wagner CL. Maternal vitamin D insufficiency and risk of adverse pregnancy and birth outcomes: A systematic review and meta-analysis of longitudinal studies. PLoS One. 2017;12(3):e0173605.
38Haider BA, Bhutta ZA. Multiple-micronutrient supplementation for women during pregnancy. Cochrane Database Syst Rev. 2017 Apr 13;4(4):Cd004905.
39Das JK, Hoodbhoy Z, Salam RA, Bhutta AZ, Valenzuela-Rubio NG, Prinzo ZW, Bhutta ZA. Lipid-based nutrient supplements for maternal, birth, and infant developmental outcomes. Cochrane Database of Systematic Reviews. 2018 (8).
40Hofmeyr GJ, Lawrie TA, Atallah Á N, Torloni MR. Calcium supplementation during pregnancy for preventing hypertensive disorders and related problems. Cochrane Database Syst Rev. 2018 Oct 1;10(10):Cd001059.
41Abraha I, Bonacini MI, Montedori A, Di Renzo GC, Angelozzi P, Micheli M, et al. Oral iron-based interventions for prevention of critical outcomes in pregnancy and postnatal care: An overview and update of systematic reviews. J Evid Based Med. 2019 May;12(2):155-66.
42Bourassa MW, Osendarp SJM, Adu-Afarwuah S, Ahmed S, Ajello C, Bergeron G, et al. Review of the evidence regarding the use of antenatal multiple micronutrient supplementation in low- and middle-income countries. Ann N Y Acad Sci. 2019 May;1444(1):6-21.
43Palacios C, Kostiuk LK, Peña-Rosas JP. Vitamin D supplementation for women during pregnancy. Cochrane Database Syst Rev. 2019 Jul 26;7(7):Cd008873.
44Adu-Afarwuah S. Impact of nutrient supplementation on maternal nutrition and child growth and development in Sub-Saharan Africa: the case of small-quantity lipid-based nutrient supplements. Matern Child Nutr. 2020 Dec;16 Suppl 3(Suppl 3):e12960.

45Lassi ZS, Padhani ZA, Rabbani A, Rind F, Salam RA, Das JK, Bhutta ZA. Impact of Dietary Interventions during Pregnancy on Maternal, Neonatal, and Child Outcomes in Low- and Middle-Income Countries. Nutrients. 2020 Feb 19;12(2).
46Lassi ZS, Padhani ZA, Rabbani A, Rind F, Salam RA, Bhutta ZA. Effects of nutritional interventions during pregnancy on birth, child health and development outcomes: A systematic review of evidence from low-and middle-income countries. Campbell Systematic Reviews. 2021;17(2):e1150.
47Daly M, Kipping RR, Tinner LE, Sanders J, White JW. Preconception exposures and adverse pregnancy, birth and postpartum outcomes: Umbrella review of systematic reviews. Paediatr Perinat Epidemiol. 2022 Mar;36(2):288-99.
48Carducci B, Keats EC, Bhutta ZA. Zinc supplementation for improving pregnancy and infant outcome. Cochrane Database Syst Rev. 2021 Mar 16;3(3):Cd000230.
49Zhao R, Zhou L, Wang S, Yin H, Yang X, Hao L. Effect of maternal vitamin D status on risk of adverse birth outcomes: a systematic review and dose-response meta-analysis of observational studies. Eur J Nutr. 2022 Sep;61(6):2881-907.
50Ciulei MA, Smith ER, Perumal N, Jakazi CS, Sudfeld CR, Gernand AD. Nutritious Supplemental Foods for Pregnant Women from Food Insecure Settings: Types, Nutritional Composition, and Relationships to Health Outcomes. Curr Dev Nutr. 2023 Jun;7(6):100094.
51Hansen R, Sejer EPF, Holm C, Schroll JB. Iron supplements in pregnant women with normal iron status: A systematic review and meta-analysis. Acta Obstet Gynecol Scand. 2023 Sep;102(9):1147-58.
